# Multi-Omics Approach Reveals Genes and Pathways Affected in Miller-Dieker Syndrome

**DOI:** 10.1007/s12035-024-04532-7

**Published:** 2024-11-07

**Authors:** Gowthami Mahendran, Kurtis Breger, Phillip J. McCown, Jacob P. Hulewicz, Tulsi Bhandari, Balasubrahmanyam Addepalli, Jessica A. Brown

**Affiliations:** 1 Department of Chemistry and Biochemistry, University of Notre Dame, Notre Dame, IN 46556 USA; 2 Department of Chemistry, University of Cincinnati, Cincinnati, OH 45221 USA; 3 Present Address: Department of Internal Medicine, Division of Nephrology, Michigan Medicine, University of Michigan, Ann Arbor, MI 48109 USA

**Keywords:** MDS, Gene expression, DEGs, Transcriptomics, Proteomics, METTL16, mTOR

## Abstract

Miller-Dieker syndrome (MDS) is a rare neurogenetic disorder resulting from a heterozygous deletion of 26 genes in the MDS locus on human chromosome 17. MDS patients often die in utero and only 10% of those who are born reach 10 years of age. Current treatments mostly prevent complications and control seizures. A detailed understanding of the pathogenesis of MDS through gene expression studies would be useful in developing precise medical approaches toward MDS. To better understand MDS at the molecular level, we performed RNA sequencing on RNA and mass spectrometry on total protein isolated from BJ (non-MDS) cells and GM06097 (MDS) cells, which were derived from a healthy individual and an MDS patient, respectively. Differentially expressed genes (DEGs) at the RNA and protein levels involved genes associated with phenotypic features reported in MDS patients (CACNG4, ADD2, SPTAN1, SHANK2), signaling pathways (GABBR2, CAMK2B, TRAM-1), and nervous system development (CAMK2B, BEX1, ARSA). Functional assays validated enhanced calcium signaling, downregulated protein translation, and cell migration defects in MDS. Interestingly, overexpression of methyltransferase-like protein 16 (METTL16), a protein encoded in the MDS locus, restored defects in protein translation, phosphor states of mTOR (mammalian target of rapamycin) pathway regulators, and cell migration in MDS cells. Although DNA- and RNA-modifying enzymes were among the DEGs and the intracellular SAM/SAH ratio was eightfold lower in MDS cells, global nucleoside modifications remained unchanged. Thus, this study identified specific genes and pathways responsible for the gene expression changes, which could lead to better therapeutics for MDS patients.

**Supplementary Information:**

The online version contains supplementary material available at 10.1007/s12035-024-04532-7.

## Introduction

The human brain is an integrated network of billions of neuronal cells. The precise organization in forming this intricate neuronal network requires a proper coordination of neurogenesis, synaptic pruning, synaptogenesis, and neuronal migration [1]. The human chromosome 17p13.3 region is a genomically unstable region linked to various neurodevelopmental diseases [2]. Miller-Dieker syndrome or MDS (OMIM 247200), which is classified as a rare neurogenetic disease by the National Institutes of Health (NIH) and Genetic and Rare Diseases Information Center (GARD), is a severe grade of lissencephaly, which refers to an unusually smooth brain in humans, that entails a critical gyral (i.e., ridges on the surface of cerebral cortex) malformation (agyria) due to neuronal migration failure (pachygyria) [3]. In general, the life expectancy of MDS patients is related to the severity of the lissencephaly. MDS patients often die in utero at 10–20 weeks of post-gestation. However, children who are born display the following phenotypes: lissencephaly, developmental delay due to severe postnatal growth retardation [4], reduced lifespan [5–7], distinctive craniofacial features (e.g., microcephaly [8], smaller jaw [9], upturned nares [10], prominent forehead [11], high arched palate [12]), ventriculomegaly (enlarged brain ventricles) [10], low muscle tone, aspiration pneumonia [13], and severe neurological abnormalities [14] that can result in intellectual disabilities, seizures, and/or epilepsy [15]. Besides severe mental defects, other related congenital abnormalities may affect the heart (e.g., enhanced hypertrophy, ventricular septal defect [16]), gastrointestinal, and/or kidney functionalities (e.g*.*, multicystic dysplastic kidney [10]). Treatment plans are based on the symptoms each person experiences, primarily aiming to prevent complications and control seizures [17]. The most common cause of death is aspiration pneumonia, caused by swallowing and breathing difficulties [13]. MDS is a complicated neurogenetic disorder that requires a broader knowledge of all associated pathways at the molecular level, most especially the anomalies affecting cardiac function, organ development, and motor regulation.

**Fig. 1:**
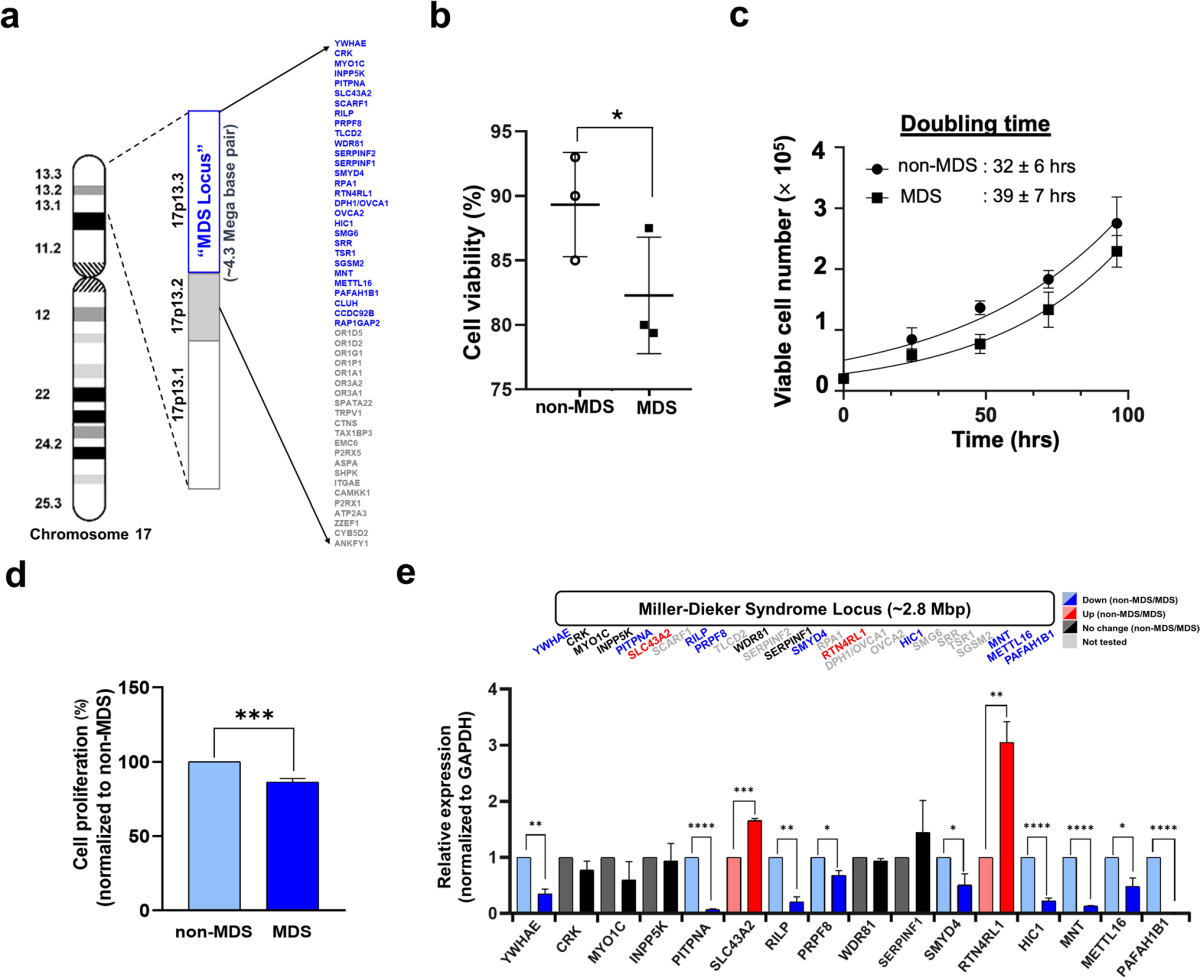
Effect of gene deletion within and near the MDS locus in MDS cells. **a** Schematic illustration of the gene deletion site at the MDS locus (blue) in human chromosome 17p13.3 and 17p13.2 (gray). Gene names are listed at the far right. GM06097 cells (i.e., MDS cells) have genes deleted in the entire MDS locus as well as part of 17p13.2 for a total of 51 genes in a 4.3-Mbp region. Gene order was obtained from the Human Genome Project Ensemble Database. Schematics are not drawn to scale. **b** Trypan blue exclusion assay was performed to analyze the cell viability of non-MDS and MDS cells. **c** Doubling time of both cell types were determined in their log phase of growth using nonlinear regression. **d** Cellular proliferation of non-MDS and MDS cells was tested using an MTT assay. **e** RT-qPCR was used to measure expression levels of 16 out of 26 genes in the MDS locus. Expression levels are denoted as upregulated (red) or downregulated (blue) with respect to the non-MDS value set at 1. All RNAs were normalized to the housekeeping gene, GAPDH. RT-qPCR values are averages of three independent experiments ± standard deviation. *p* values were calculated using a two-tailed unpaired Student’s *t* test. ****p value < 0.0001, ***p value < 0.0002, **p value < 0.0021, *p value < 0.033, ^ns^*p* value < 0.1234

At the genetic level, MDS is defined by the haploinsufficiency of 26 protein-coding genes (~ 2.8-Mbp region) encoded in the MDS locus on human chromosome 17p13.3 (Fig. 1a) [5]. This deletion is linked to the haploinsufficiency of two pivotal genes: *PAFAH1B1* and *YWHAE*, which represent the boundaries of the MDS locus (Fig. 1a) [6] and are involved in neuronal migration. PAFAH1B1 (also known as LIS1) is essential in early embryonic development, as this protein contributes to the proper neuronal migration in the adult brain to form several regions organized into laminar structures [7]. YWHAE (also known as 14–3-3ε) regulates neuronal migration by binding to the LIS1-interacting protein NDEL1 (NudE neurodevelopment protein 1 like 1), a protein that aids in mitotic spindle assembly [8]. As part of the cytoplasmic dynein complex, both PAFAH1B1 and YWHAE, together with NDEL1, control neuronal migration through cell division and contribute to the “small brain” phenotype in an MDS forebrain-type organoid system via weakened β-catenin/Wnt signaling [18, 19]. Neuronal migration speed and displacement can be rescued upon restoration of PAFAH1B1 and YWHAE levels [20]. PAFAH1B1 haploinsufficiency alone is not adequate to cause the MDS severities reported, suggesting that other MDS-locus genes or perturbations to genes outside of the MDS locus are involved in disease progression [21]. Indeed, other MDS-locus genes are involved in various signaling and metabolic pathways (*YWHAE:* signal transduction pathways [22], *CRK*: signal transduction [23], *PITPNA*: Inositol lipid signaling [24], *RPA1:* DNA metabolism [25], *METTL16*: methionine cycle [26]) emphasizing the importance of studying gene deletions in MDS (Fig. 1a).

Herein, we leveraged MDS patient-derived skin fibroblasts (GM06097) to investigate the gene expression changes at the RNA and protein levels. While more genes were altered at the RNA level (~ 2800 differentially expressed genes (DEGs)) than the protein level (~ 450 DEGs), our multi-omics results and pathway analyses uncovered significantly enriched genes associated with key regulatory pathways, including synaptogenesis, cardiac hypertrophy signaling, STAT3 signaling, and EIF2 signaling, which may contribute to dysfunctions of the brain, heart, and other vital organs [27]. Interestingly, reduced expression of the MDS locus-encoded gene methyltransferase-like protein 16 (METTL16) contributed to slower cell migration, global protein translation, and intracellular levels of *S*-adenosylmethionine (SAM). Moreover, we show that global protein and SAM levels depend on the mTOR pathway, whereby the phosphor states of the mTOR pathway downstream regulators (p-mTOR, p-P70 S6K, p-4e-BP1) are decreased in MDS cells. Thus, this study provides insights at the molecular level, allowing us to propose that various FDA-approved therapeutics could be considered for treating MDS patients while better therapeutic strategies can be developed.

## Materials and Methods

### Cell Culture

Fibroblast cells isolated from healthy individuals and an MDS patient were used in this study. BJ cells (ATCC (CRL-2522) [28], RRID: CVCL_3653) derived from a < 1-month-old male neonate’s foreskin fibroblasts were used as control and herein referred to as non-MDS cells. Two MDS cell lines (GM06047 cells, RRID: CVCL_5U19 and GM06097 cells, RRID: CVCL_5U20 purchased from Coriell Institute for Medical Research) were derived from a 4-month-old male’s fibroblasts and a 1-year-old female’s skin fibroblasts, respectively, were used as a disease model. Confirmation of the deletion in chromosome 17 was established by the Coriell Institute for Medical Research [29, 30]. BJ and GM06097 cells were cultured in complete Minimal Eagle Medium (MEM, Gibco) supplemented with 10% fetal bovine serum (FBS), 2 mM glutamate, and a solution of 1 × penicillin–streptomycin. GM06047 cells were cultured in 1:1 ratio of complete MEM and Dulbecco Minimal Eagle Medium (DMEM, Gibco) supplemented with 15% FBS, 2 mM glutamate, and a solution of 1 × penicillin–streptomycin. Cells were allowed to grow until they reached nearly 80–90% confluency at 37 °C and 5% CO_2_. Media replenishment was completed every 2 to 3 days. Cells were detached from culture plates using 1 × TrypLE Express with phenol red (Gibco). Subcultivation ratios were 1:2 to 1:9 for BJ, 1:5 for GM06097, and 1:3 for GM06047.

### Trypan Blue Exclusion and MTT Assays

Cell viability was tested using the trypan blue exclusion assay. Non-MDS and MDS cells were seeded at a density of 5 × 10^4^ cells/well in a 24-well plate. Viable cell counting was performed using trypan blue after 24 h of seeding. To determine the doubling time, cell viability of non-MDS and MDS cells was counted at different time intervals (24, 48, 72, 96 h). Using GraphPad Prism (version 8.0.1), an exponential growth equation (Eq. 1) was applied to extrapolate the rate constant.

1 $$Y ={{Y}_{0} e}^{kX}$$

*Y* is the viable cell number, *Y*_0_ is the initial seeding density, *k* is the rate constant, and *X* is time. Doubling time was calculated as follows: ln (2)/*k*. Proliferation of non-MDS and MDS cells was assessed using MTT (3-(4,5-Dimethylthiazol-2-yl)-2,5-Diphenyltetrazolium Bromide) cell proliferation reagent (Thermo Fisher Scientific). Non-MDS and MDS cells were seeded in a 96-well plate at 6000 cells/well in 200 μl complete MEM medium. On the following day, cells were treated with 10 μl of 5 mg/ml MTT for 4 h at 37 °C in the dark. Once the purple precipitate was visible, cells were treated overnight with 10% SDS at 37 °C. Absorbance at 590 nm was measured the following day using a Synergy|H1 microplate reader (Agilent BioTek Gen5 microplate reader and imager software)*.* Cell metabolic activity of the non-MDS cells was normalized to 1, and the activity of MDS cells was reported relative to the activity of non-MDS cells. Three biological replicates were performed.

### RNA Sequencing (RNA-Seq) and Data Analysis

**Fig. 2:**
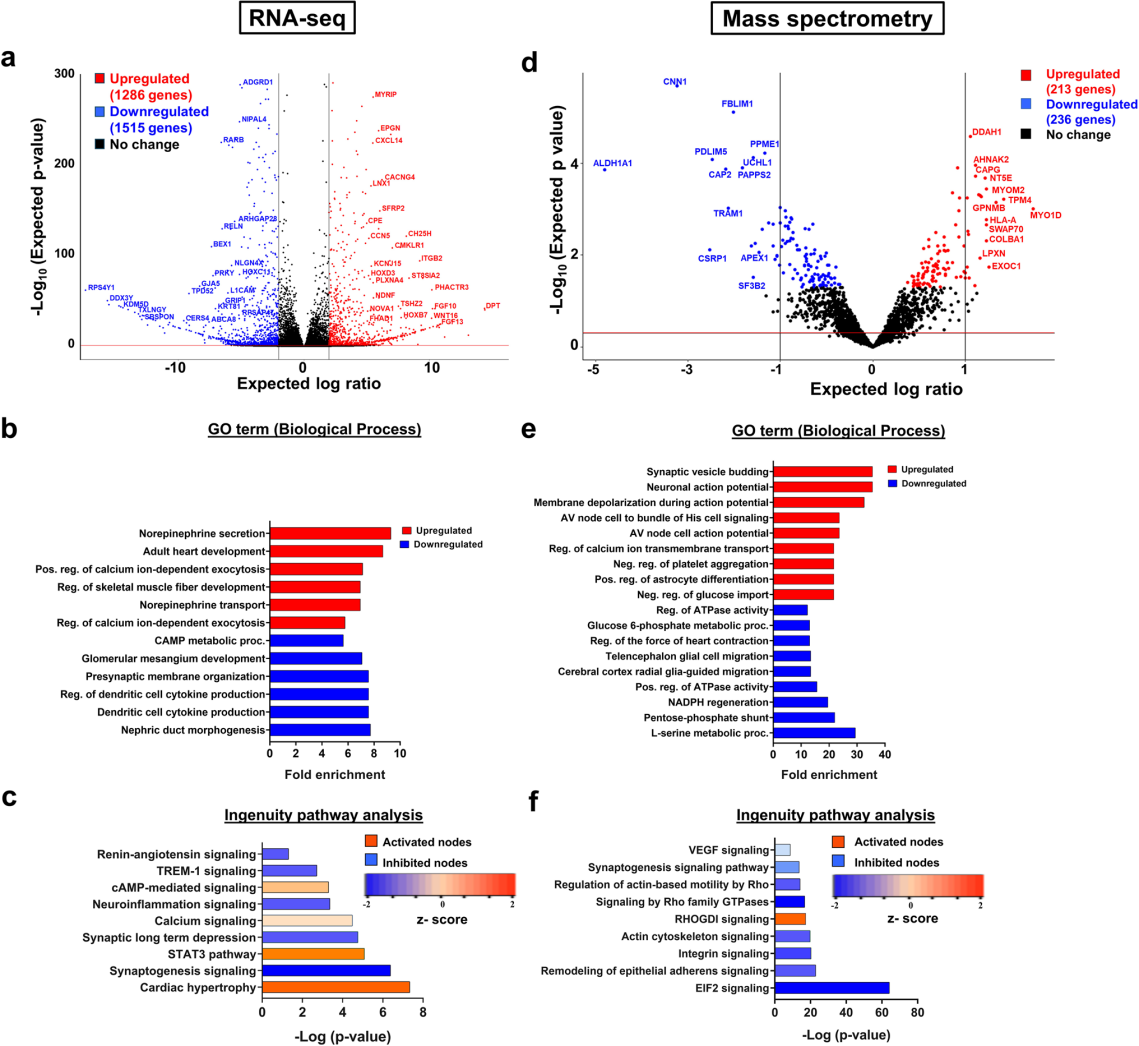
Differential expression analyses of transcriptomics and proteomics studies. **a** Volcano plot of DEGs from RNA-seq using − 0.5 > Expected log ratio > 0.5 (represented by the black vertical lines) and -log (expected *p* value) > 1.3 (represented by the red horizontal lines) cutoffs. At the RNA level, 1286 genes were upregulated (red), and 1515 genes were downregulated (blue) in MDS relative to non-MDS cells. RNA-seq was performed using four biological replicates (*n* = 4). **b** GO term analysis of transcriptomics results for biological processes (BP). Fold enrichment of DEGs in their associated pathways is shown as a bar plot. **c** Qiagen IPA analysis of canonical pathways at the RNA level identified enhanced cardiac hypertrophy and repressed synaptogenesis in MDS. Activated nodes (upregulated) are orange (*z* score ≥ 2) and inhibited nodes (downregulated) are blue (*z* score ≤  − 2). Color intensity is based on the magnitude of the *z* score values. **d** Volcano plot of DEGs from MS/MS study using − 1 > Expected log ratio > 1 (represented by the black vertical lines) and -log (expected *p* value) > 1.3 (represented by the red horizontal line) cutoffs. At the protein level, 213 genes were upregulated (red), and 236 genes were downregulated (blue) in MDS relative to non-MDS cells. MS/MS was performed using five biological replicates (*n* = 5). **e** DEGs from MS/MS were analyzed using Shiny GO to determine the significant changes in biological processes (BP). Fold enrichment of DEGs in their associated pathways is shown as a bar plot. **f** Qiagen IPA analysis of canonical pathways at protein level identified repressed EIF2 signaling. Activated nodes are orange (*z* score ≥ 2) and inhibited nodes are blue (*z* score ≤  − 2). Color intensity is based on the magnitude of the *z* score values

Four biological replicates of non-MDS and MDS cells were harvested (4 × 100-mm tissue culture-treated plates) at 80% confluency. RNeasy kit (Qiagen 74104) was used to extract total RNA for Illumina HiSeq 2000 sequencing at the Notre Dame Genomics & Bioinformatics Core Facility. NEB-Next Poly(A) mRNA Magnetic Isolation kit (NEB #E3370) was utilized for intact poly(A)+ RNA isolation from the extracted total RNA. RNA concentration was determined using a Qubit 2.0 fluorometer, and RNA quality was assessed using the Agilent 2100 Bioanalyzer. All RNA samples had a 28S/18S rRNA ratio > 1 and RNA integrity number > 8. Raw reads were obtained (approximately 60–70 × 10^6^ reads by paired-end sequencing for each replicate) and subjected to quality control using FastQC. The Trimmomatic software (version 0.36) was then used to remove adapters from the RNA reads and any poor-quality reads indicated by FastQC (version 0.11.5) [31]. HISAT2 (version 2.1.0) software aligned the reads to the hg38 genome [32]. Finally, Stringtie software (version v2.1.3b) was used for transcript assembly and quantification [33]. A DESeq2 R script was used within R-studio to calculate differential gene expression (− 0.5 > Expected log ratio > 0.5 and -log (expected *p* value) > 1.3) and to create a volcano plot (see Fig. 2a) [34]. A complete list of DEGs is in Supplementary file 1.

### Mass Spectrometry for Proteomics

Whole-cell protein lysates were prepared using ~ 4 million non-MDS and MDS cells harvested at 80–90% confluency. Cell pellets were resuspended in an equal volume of RIPA buffer (50 mM Tris pH 7.5 at room temperature, 150 mM NaCl, 5 mM EDTA, 2% Triton X-100, 0.2% sodium deoxycholate, 0.2% SDS, 2% IGEPAL, 2 mM PMSF (American Bio), 2% protease inhibitor cocktail EDTA-free (Sigma-Aldrich) and NET buffer (50 mM Tris pH 7.5 at room temperature, 150 mM NaCl, and 5 mM EDTA). After 30-min incubation on ice, lysates were centrifuged at 12,000 rpm for 20 min at 4 °C. Protein concentration was quantified using a BCA assay kit (Thermo Fisher Scientific) according to manufacturer’s protocol. For each cell line, five samples of 10 µg/µl total protein were injected into a Thermo-Finnegan Q-Exactive HF (QEHF) mass spectrometer coupled to a Waters M-Class ultrahigh pressure liquid chromatography system via a nano-electrospray ionization source. Mass spectra were collected by the Orbitrap, and the tandem mass spectra were generated. Select peptide ion fragmentation was achieved through high energy collisional dissociation. To identify proteins, Proteome Discoverer 2.2 software (Thermo Fisher Scientific) using Mascot search engine with a decoy search at a 1% false discovery rate was used to match the tandem mass spectra with peptides expected for proteins in the SwissProt Database [35]. Differential enrichment analysis of proteomics data (DEP) package (Bioconductor) in R-studio was used to analyze differential protein expression and to create a volcano plot (see Fig. 2d) using -log (expected *p* value) > 1.3 and − 1 > expected log ratio > 1 as cutoffs. The output from DEP is provided in Supplementary file 2.

### DEG Analysis of Transcriptomics and Proteomics Data Using GO, KEGG, and *IPA* Software Tools

Significant enrichment of Gene Ontology (GO) terms and Kyoto Encyclopedia of Genes and Genomes (KEGG) pathways were assessed using the Fisher’s exact test in ShinyGO 0.76.3 tool using False-discovery rate (FDR) ≤ 0.05 and *p* < 0.05 as cutoffs [34] (Supplementary files 3 and 4). For Qiagen IPA software, DEGs (ENSEMBL annotation) identified using DESeq2 for RNA and DEP for proteins were uploaded as an excel file (Supplementary files 1 and 2) [36]. IPA provides a graphical summary of related pathways, altered top canonical pathways, involved upstream regulators, associated diseases, and functions showing the link between molecules identified to understand the pathway responses and its functionalities.

### RT-qPCR

Non-MDS and MDS cell pellets (1 × 10^6^ cells/well in a 100-mm plate) were lysed using Trizol (Life Technologies), and RNA was isolated per the manufacturer’s protocol. RNA pellets were resuspended in RNase-free water. RNA concentration was determined using a NanoDrop™ One^C^. DNase treatment was performed on the extracted RNA using RQ1 DNase (Promega) per the manufacturer’s protocol and the RNA was cleaned up using Trizol. The first-strand cDNA synthesis was performed using SuperScript III reverse transcriptase (Invitrogen) and random hexamer primers (Thermo Fisher Scientific). Primer pairs (Sigma-Aldrich, Table S1) for gene targets were designed using Primer-BLAST (NCBI) [37] considering all transcript variants of a gene. FastStart SYBR Green Master Mix (Sigma-Aldrich) was used, and all runs included negative controls (i.e., no template and no reverse transcriptase controls). Optimal cycling conditions using a StepOnePlus Real-Time PCR System (Applied Biosystems) were as follows: denaturation: 95 °C for 10 min, annealing: 62 °C for 1 min, and extension: 72 °C for 1 min. The 2^−ΔΔϹt^ values were calculated for each gene-of-interest (GOI) from individual ΔCt values with normalization to the housekeeping gene, GAPDH. Three biological replicates were performed, including three technical replicates for each GOI.

### Western Blot

Total protein (30 µg) was separated using 10% Mini-PROTEAN precast gels (Bio-Rad) at 200 V for 2 h at room temperature. Proteins were transferred in a 1 × transfer buffer (25 mM Tris base, 192 mM glycine, 20% methanol) to a nitrocellulose membrane at 115 V for 2 h at 4 °C. The membrane was blocked with 5% non-fat dry milk for an hour followed by PBST washes (PBS and 1% Tween-20). The membrane was then probed using the relevant primary antibody (see Table S2 for source and dilution information) overnight at 4 °C and fluorescently labeled secondary antibodies ((goat anti-rabbit IgG highly cross-adsorbed secondary antibody (Alexa Fluor™ Plus 555, Fisher Scientific)) and goat anti-mouse IgG highly cross-adsorbed secondary antibody (Alexa Fluor Plus 647, Fisher Scientific) (see Table S3 for source and dilution information) were added the following day to visualize the proteins using an Azure c400 with channels for Cy3 (*λ*_ex_ = 555 nm) and Cy5 (*λ*_ex_ = 630 nm). Relative protein levels were calculated using Image Studio Lite™ image processing software (*LI-COR,* RRID:SCR_013715) [38] by quantitating the band intensities as a ratio of MDS to non-MDS cells normalized to the total protein using Ponceau S staining.

### cAMP and Calcium Level Analysis Using cAMP Immunoassays

Cyclic adenosine monophosphate (cAMP) levels, which indirectly correspond to calcium levels, in MDS and non-MDS cells were quantified using a cAMP Direct Immunoassay Kit (Abcam), albeit with slight modifications in the initial steps. Non-MDS and MDS cells were seeded on 100-mm culture plates to harvest 2 × 10^5^ cells. The experiment was conducted with three biological triplicates. After cell lysis, the cAMP assay procedure was carried out according to the manufacturer’s instructions.

### Subcellular Fractionation Assay

Three 100-mm culture plates of non-MDS and MDS cells were washed with PBS and were trypsinized. Cells were spun down at 1000 rcf for 5 min at 4 °C. The cell pellet was resuspended in equal volumes of NET buffer (50 mM Tris pH 7.5 at room temperature, 150 mM NaCl, and 5 mM EDTA) and an equal volume of cytoplasmic lysis buffer (NET buffer, 1% IGEPAL, 2 mM PMSF). The cell suspension was incubated on ice for 5 min. The lysis efficiency was checked under the microscope before centrifuging at 1000 rcf for 5 min at 4 °C. The cytoplasmic fraction was collected. The pellet of nuclei was washed gently with NET buffer and centrifuged at 1000 rcf for 5 min at 4 °C. Nuclei were resuspended in 100 µl NET buffer and then in 400 µl RIPA buffer (50 mM Tris pH 7.5 at room temperature, 150 mM NaCl, 5 mM EDTA, 2% Triton X-100, 0.2% sodium deoxycholate, 0.2% SDS, 2% IGEPAL, 2 mM PMSF (American Bio), 2% protease inhibitor cocktail EDTA-free (Sigma-Aldrich). Cells were sonicated (three times for 7 s at 30-s intervals) on ice and were centrifuged at maximum rpm for 20 min at 4 °C. The nuclear fraction was collected. Cytoplasmic and nuclear fractions were run on 10% SDS PAGE gels. Western blot was performed as described in said section.

### Plasmids

Human METTL16 cDNA (Material No: MHS6278-202,759,933, clone ID: 6,174,508) was purchased from GE Healthcare Dharmacon. PCR primers (Keck Oligo Synthesis Resource, Yale University) were designed to amplify the *METTL16* gene (region encoding amino acids 1–562). In addition, the forward primer contained the nucleotide sequence for the *HindIII* restriction site and a 1× FLAG tag while the reverse primer had the nucleotide sequence for the stop codon and a *Not1* restriction site. The digested fragments were then inserted into a pcDNA3 plasmid at the *HindIII* and *NotI* sites. Restriction digests and DNA sequencing (Keck DNA Sequencing Core, Yale University) were used to confirm the plasmid contained the *METTL16* gene. The catalytically inactive R82A/N184A mutant was created using two rounds of site-directed mutagenesis per the manufacturer’s protocol (QuikChange). DNA sequencing confirmed mutation sites. The resulting plasmids include wild-type (WT) FLAG-METTL16 (amino acids 1–562) and catalytically inactive FLAG-dMETTL16 (amino acids 1–562, R82A/N184A). Primers for cloning and site-directed mutagenesis are provided in Table S4.

### SUnSET Assay

Non-MDS and MDS cells, transfected with an empty pcDNA3 vector (EV), WT FLAG-METTL16, and FLAG-dMETTL16, were seeded at a density of 1 × 10^5^ cells/well in a 12-well plate and were allowed to grow until reaching 80–90% confluency in antibiotic-depleted media. The media was changed 1 h prior to the puromycin treatment. Cells were treated with 8 µg/ml of puromycin (Sigma-Aldrich) for 30 min. After a quick wash with ice cold PBS, cells were lysed, and the total protein was extracted according to the method described above for western blots. Proteins were separated on 10% SDS-PAGE gels, and the membrane was probed overnight using anti-puromycin antibody (EMD Millipore, Table S2). After washing, an HRP-conjugated secondary antibody (Sigma-Aldrich, Table S3) was added and was incubated at room temperature for an hour. Clarity Western ECL Substrate (Bio-Rad) was applied to the membrane, and the chemiluminescent signal was detected using an Azure c400. Band intensities were quantified using Image Studio Lite software (*LI-COR*, version 5.5). For testing the involvement of mTOR pathway, cells were treated with 250 mM Torin 1 (EMD Millipore), an mTOR pathway inhibitor, for 6 h, followed by a 30-min incubation in the presence of 8 µg/ml puromycin. Cells were then harvested, and the total protein was extracted and analyzed via an anti-puromycin western blot.

### Quantitation of SAM/SAH Levels

*S*-adenosyl methionine (SAM) and *S*-adenosyl homocysteine (SAH) levels in non-MDS and MDS cells were measured using a SAM/SAH ELISA combo kit (Cell Biolabs: MET-5151-C) according to the manufacturer’s instructions (1 × 10^6^ cells in a 100-mm plate). The absorbance was measured at 450 nm using Synergy|H1 microplate reader (Agilent BioTek Gen5 microplate reader and imager software). SAM and SAH levels in non-MDS and MDS cells were determined using a standard curve, and the assay was performed in biological triplicates. METTL16 was overexpressed by transfecting cells (1 × 10^5^ cells/well on a 12-well plate) with 1 µg/ml pcDNA3 vector encoding WT or mutant N-terminal FLAG-tagged METTL16 using Human Dermal Fibroblast (HDF) Avalanche Transfection Reagent (EZ Biosystems) in antibiotic-depleted media according to manufacturer’s instructions. As a control, non-MDS and MDS cells received an empty pcDNA3 vector (EV) (1 µg/ml). For testing the involvement of mTOR pathway, cells were treated 48 h after transfection with 250 mM Torin 1 (EMD Millipore) for 6 h. Untreated and Torin 1-treated cells were then harvested, and cell lysates were extracted according to the manufacturer’s protocol for the SAM/SAH analysis.

### Quantitation of Nucleoside Modifications

Five replicates of total RNA from the non-MDS and MDS cells were obtained using Trizol per the manufacturer’s protocol. Then 1 µg of RNA from each sample was digested to nucleosides as previously described [39]. Nucleosides were analyzed using ultra high-performance liquid chromatography-tandem mass spectrometry (UHPLC-MS/MS). First, nucleoside separation was performed using an ultra-high-performance liquid chromatography (UHPLC) instrument (Vanquish UHPLC, Thermo Fisher Scientific) equipped with a Waters Acquity HSS T3 column (1 × 100 mm, 1.8 μm). Reverse phase chromatography was performed following the LC conditions as previously described [40]. Mobile phase A (MPA, 5.3 mM ammonium acetate at pH 4.5) and mobile phase B (MPB, 60% MPA + 40% acetonitrile (ACN)) were run as a gradient: 0% MPB (from 0 to 7.6 min), 2% MPB at 15.7 min, 3% MPB at 19.2 min, 5% MPB at 25.7 min, 25% MPB at 29.5 min, 50% MPB at 32.3 min, 75% MPB at 36.4 min to 36.6 min, 99% MPB at 39.6 min to 46.8 min, then returning to 0% MPB at 46.9 min. Flow rate was 0.1 ml/minute and the column temperature was 30 °C. Samples were resuspended in MPA before injection. A triple quadrupole mass spectrometer (TSQ Quantiva, Thermo Fisher Scientific) was used for detection. Data was acquired in positive ion mode (3500 V) with electro-spray ionization (ESI). Sheath gas, auxiliary gas, and sweep gas were 45, 10, and 0 arbitrary units respectively; ion transfer tube temperature was 290 °C; and vaporizer temperature was 100 °C.

All five replicates for the non-MDS and MDS samples were analyzed back-to-back in a continuous instrument run. Data processing was completed with Xcalibur 3.0 software using the retention time (min), molecular ion m/z ([MH +]), and fragment ion m/z ([BH2 +]). Extracted ion chromatograms (EIC) were prepared for each nucleoside and the peak areas were determined. EIC peak areas for nucleosides were integrated and normalized with total peak area for all canonical RNA nucleosides (A, C, G, and U). Normalized values were then averaged for both MDS and non-MDS samples. The fold change of the averaged MDS signal was determined relative to the averaged non-MDS signal. RNA nucleosides that we examined are listed in Table S5. Detailed output excel file can be found in Supplementary file 5 (“ND” denotes that signal was not detected).

### Wound-Healing Assay

METTL16 was overexpressed by transfecting MDS cells (5 × 10^4^ cells/well on a 96-well plate) with 1 µg/ml pcDNA3 vector encoding WT or mutant N-terminal FLAG-tagged METTL16 using Human Dermal Fibroblast (HDF) Avalanche Transfection Reagent (EZ Biosystems) in antibiotic-depleted media according to the manufacturer’s instructions. As a control, non-MDS were untransfected and MDS cells received an empty pcDNA3 vector (EV) (1 µg/ml). Wounds were created using a 10-µl pipette tip, followed by rinsing with PBS to eliminate residual floating cells. Fresh media was added, and the gap width was imaged at 0, 6, 12, 24, and 30 h to observe wound closure. All images were captured with a 10 × objective on an Eclipse TE2000 inverted microscope (Nikon) with a Hamamatsu CMOS camera connected to NIS-Elements BR 413.04 64-bit software (Nikon). The gap width was measured at each time point, and the images were processed using ImageJ 1.54f (RRID:SCR_003070).

### Statistical Analysis

Values for quantitative assays are averages of three independent experiments ± standard deviation. Statistical significance was calculated via a two-tailed unpaired Student’s *t* test or two-way ANOVA using GraphPad Prism 8.0.1 software (RRID:SCR_002798). Statistical significance was defined as *p* values ≤ 0.05 (****p value < 0.0001, ***p value < 0.0002, **p value < 0.0021, *p value < 0.033, ^ns^*p* value < 0.1234).

## Results

### MDS Cells Exhibit Decreased Cell Viability and Proliferative Ability

To investigate gene expression changes that occur due to MDS, we began with cell lines that have been used previously for MDS-focused studies [19–21]: BJ cells as the control (hereafter referred to as non-MDS cells) and two MDS cell lines known as GM06047 and GM06097 [29, 30]. All three cell lines are fibroblasts derived from patients at a relatively similar age (~ 1 to 12 months old) [21, 28–30]. Additionally, the source of fibroblasts is similar for GM06097 (skin, arm) and BJ (foreskin) [21]. GM06047 cells were derived from an MDS patient having a ring chromosome as a result of the heterozygous deletion on chromosome 17p13.3 [20]. These cells exhibited poor growth; therefore, they were excluded from further study. GM06097 cells (hereafter referred to as MDS cells) were derived from an MDS patient who had a 4.3-Mbp heterozygous gene deletion [19] so that genes *YWHAE* through *ANKFY1* are missing on one copy of chromosome) [20]. These cells have a deletion similar to a 4-year-old MDS patient whose pathology and symptoms underwent extensive clinical evaluation, which may be helpful in establishing genotype–phenotype relationships [41]. When culturing cells, we observed that non-MDS cells formed more dense and shorter dendrite-like extensions whereas MDS cells tended to form more sparsely distributed and elongated dendrite-like extensions when observed under the light microscope (Fig. S1). In addition, MDS cells exhibited slower growth, requiring more time to reach confluency than the non-MDS cells. Therefore, cell doubling time and proliferation differences were quantified using a trypan blue exclusion cell viability assay and an MTT assay, respectively. Cell viability counting showed that MDS cells are ~ 8% less viable than non-MDS cells (Fig. 1b), and that MDS cells have a longer doubling time (39 ± 7 h) than the non-MDS cells (32 ± 6 h) (Fig. 1c). The MTT assay confirmed the slower proliferative ability in MDS cells (~ 15%) when compared to the non-MDS cells (Fig. 1d). Next, we sought to determine if the non-MDS and MDS cells would be an appropriate model system to reveal changes in gene expression. Thus, we performed RT-qPCR to quantify expression levels of genes deleted at the MDS locus because one might expect genes encoded in the MDS locus to exhibit reduced expression in MDS cells due to the heterozygous deletion. We selected 16 genes, and nine of those genes were expressed at statistically significantly lower levels in MDS than non-MDS cells (Fig. 1e). Altogether, the differences observed with respect to cellular morphology, viability/doubling time, proliferation activity, and mRNA levels suggested that BJ (non-MDS) and GM06097 (MDS) cells are a reasonable model for probing MDS at the molecular level.

### RNA-Seq and Mass Spectrometry Approaches Reveal DEGs Related to MDS

Next, we sought to identify DEGs in the transcriptome and proteome using RNA-seq and mass spectrometry, respectively. First, RNA-seq was performed on poly(A)-selected RNA isolated from non-MDS and MDS cells. DEseq2 determined that 1286 genes were upregulated, and 1515 genes were downregulated in MDS versus non-MDS cells (Fig. 2a). Among the 26 genes within the MDS locus (Fig. 1a), 19 genes were downregulated, one was upregulated, and six genes did not show significant changes in expression in MDS cells (Supplementary file 1). Functional associations of the DEGs were further investigated using GO term analysis, considering biological processes (i.e., gene products influenced by various molecular activities), cellular components (i.e., gene products related to the action of those molecular activities identified), and molecular functions (i.e., gene product activities) [42]. GO term analyses performed using up (in red) and downregulated (in blue) gene sets were highly enriched for cardiac development, various signaling pathways (i.e., calcium, cAMP, Wnt, IL2, and norepinephrine), and neuronal morphogenesis (neuroactive interactors, neuronal synaptic pathways) (Fig. 2b, Fig. S2a–b). Similar results were obtained using Kyoto Encyclopedia of Genes and Genomes (KEGG) (Fig. S2c).

Qiagen IPA software was further utilized for functional analyses and pathway studies, as this tool can be used to predict downstream effects and identify new targets or biomarkers. The graphical summary depicts the established signaling and metabolic pathways that were significantly altered (Fig. S3), most notably upregulation of cardiac hypertrophy and the STAT3 pathway as well as downregulation of synaptogenesis signaling and synaptic long-term depression (Fig. 2c). Detailed pathway information and associated genes are in Supplementary file 3. Furthermore, downregulated IL-1B (pro-inflammatory cytokine) and upregulated STAT3 in MDS were identified as central mediators (Fig. S3). As IL-1B induces the activation of STAT3 in neuronal progenitor cells to help in axon regeneration [43], the diseases and function tool of IPA were performed to reveal possible connection between STAT3 and MDS. Cancer and organismal injuries were the top two diseases and function categories that were predicted to increase, along with neurological (*HAND2*), hepatic system (*APOE*, *CACNG4*, *CAMK2B*), respiratory system (*WNT16*), and skeletal muscle developmental disorders (*ADD2*, *HAND2*) (Fig. S4a–b).

To identify gene expression changes at the protein level, bottom-up proteomics using tandem mass spectrometry (MS/MS) was employed on non-MDS and MDS whole-cell lysates. MS/MS-identified peptides and protein groups were analyzed using the PEAKS online proteomics server software [44] (Supplementary File 2), revealing 213 upregulated genes and 236 downregulated genes in MDS cells (Fig. 2d). For the heterozygous gene deletions in the GM06097 MDS cells (Fig. 1a), the proteomics results indicated that expression was altered for only six of the 26 MDS locus genes: four (YWHAE, CRK, METTL16, and PAFAH1B1) were less abundant in MDS and two (MYO1C and SERPINF1) were more abundant (Supplementary File 2). Western blot analysis validated these six DEGs from the MDS locus (Fig. S5). Next, we performed gene enrichment analyses using the GO term, KEGG, and IPA tools. GO term (Fig. 2e and S6a–b) and KEGG (Fig. S6c) analyses of the downregulated protein list suggested significant fold enrichment of proteins related to pentose phosphate pathway (PPP), which is associated with glycolysis and energy generation [45], in addition to heart and synaptic functionalities. As MDS patients often suffer from movement difficulties due to spasticity, energy generation and utilization could be compromised in these patients as a result of protein deficiencies in downstream pathways or processes. Other metabolic pathways, including amino acid metabolism, were also found to be downregulated at the protein level (Fig. 2e).

The functional and pathway analyses were corroborated by the Qiagen IPA tool (Fig. 2f, Fig. S7, Supplementary file 4), although it indicated that downregulation of EIF2 signaling (topmost) plays a major role in MDS as well as multiple dysfunctional signaling pathways (i.e., integrin, actin cytoskeletal signaling, Rho family GTPase signaling, and RHOGDI signaling). Interestingly, the downregulated epidermal growth factor (EGF) was identified by the IPA tool to be a central mediator (Fig. S7). EGF plays a vital role in cellular growth and differentiation of various cell types including neural stem cells to contribute to central nervous system (CNS) development and nerve myelination [46]. EGF and nerve growth factor (NGF) activates protein synthesis, suggesting that EIF2 signaling could be restored by EGF treatment [47]. The diseases and functions analyses by IPA (Fig. S8a–b) identified cancer and organismal injuries, similar to an analysis performed using the RNA-seq dataset (Fig. S4b), as well as compromised protein synthesis, endocrine disorders, gastrointestinal diseases, and neurological diseases. Please note, although non-MDS and MDS cells originate from individuals of different sexes, DEGs from the Y-chromosome were only 25 and 3 at the RNA and protein levels, respectively. Including these genes had no impact on pathway analyses. Altogether, our transcriptomics and proteomics analyses uncovered multiple targets to further pursue, most notably CACNG4, ADD2, NEAS, SHANK2, WNT, GABBR2, CAMK2B, TRAM-1, CAMK2B, BEX1, and ARSA.

### DEGs Are Associated with MDS Phenotypes, Vital Signaling Pathways, and Neuronal-Related Activity

**Fig. 3:**
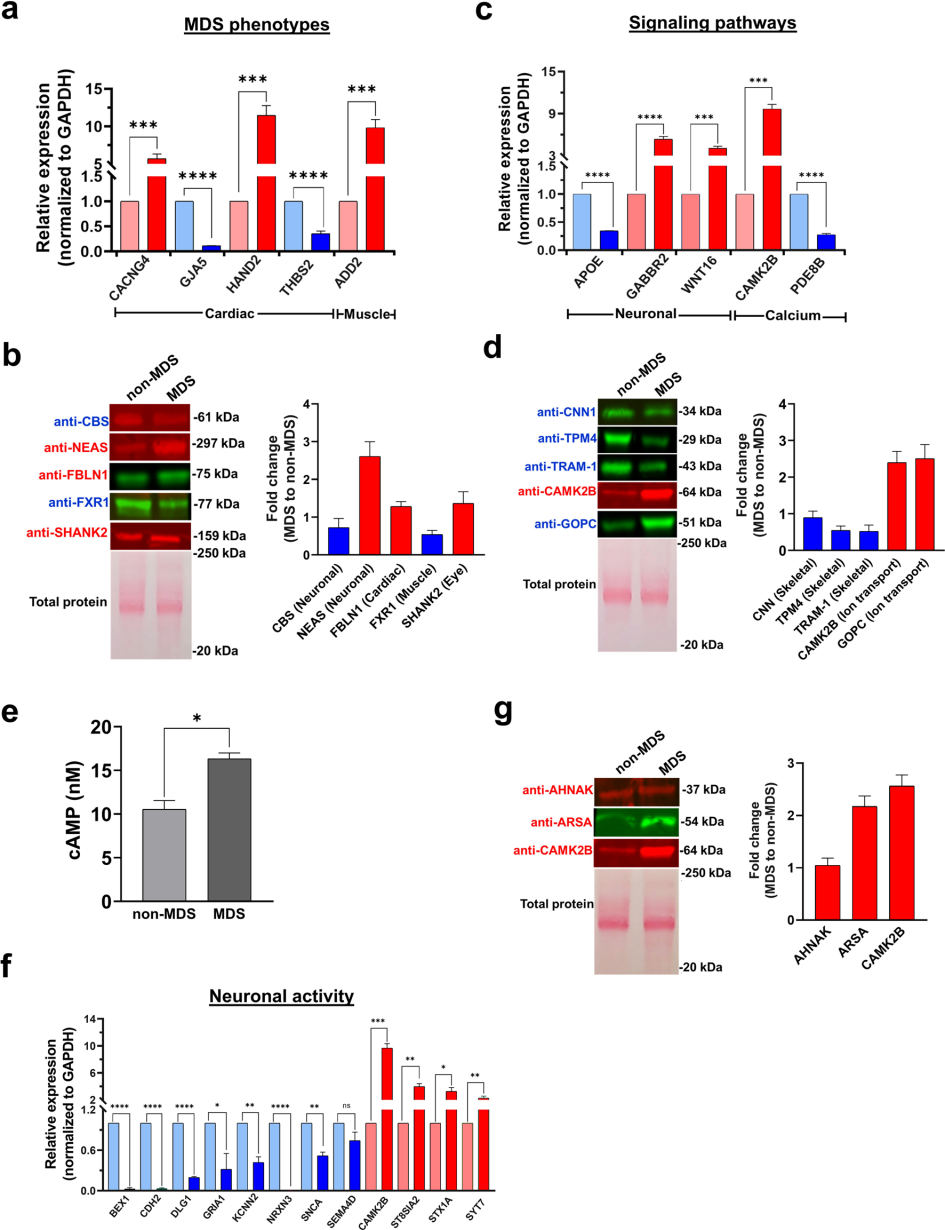
Validation of DEGs related to MDS phenotypes and various signaling pathways. **a** DEGs associated with MDS phenotypes at the RNA level were validated using RT-qPCR. **b** MDS phenotype-associated genes showing significant alterations at the protein level were validated using western blot. Similarly, DEGs associated with different signaling pathways were validated using **c** RT-qPCR and **d** western blot. **e** cAMP direct immunoassay kit was performed to determine the cAMP levels in non-MDS and MDS cells. DEGs associated with neuronal activity were validated using **f** RT-qPCR and **g** western blot. Representative western blots are next to the respective bar graphs, quantifying the fold change of protein expressed in MDS relative to non-MDS cells after total protein normalization. All quantitative values are the average ± standard deviation of biological triplicates (*n* = 3) and *p* values were calculated using a two-tailed unpaired Student’s *t* test. ****p value < 0.0001, ***p value < 0.0002, **p value < 0.0021, ^ns^*p* value < 0.1234

The DEGs identified from the transcriptomics and proteomics analyses indicated significant changes in expression of genes linked to phenotypes observed in MDS patients. To validate selected DEGs at the RNA and protein levels, we considered the list of known MDS pathologies [41] and the DEGs that appeared in perturbed pathways based on our GO, IPA, and KEGG analyses (Fig. 2, S2–4 and S6–8). At the RNA level, RT-qPCR confirmed expression changes in CACNG4 (cardiac arrythmia [48]), GJA5 (atrial fibrillation [49]), THBS2 (angiogenesis inhibitor [50]), ADD2 (membrane skeletal protein, synaptic plasticity [51]), and HAND2 (neurogenesis [52]) (Fig. 3a). At the protein level, western blots validated expression changes in CBS (mental retardation [53]), NEAS/SPTAN1 (associated with infantile epilepsy [54]), FBLN1 (cardiac morphogenesis [55]), FXR1 (muscle development [56]), and SHANK2 (neuronal differentiation in the retina [57]) (Fig. 3b).

In addition, MDS cells have notable changes in signaling pathways; therefore, we confirmed expression levels of key mRNAs using RT-qPCR (Fig. 3c): APOE (involved in multiple neuronal signaling pathways [58]), GABBR2 (gamma-aminobutyric acid signaling pathway and neuron-glial cell signaling [59]), WNT16 (wnt/β-catenin signaling to promote neurogenesis [60]), CAMK2B (calcium/calmodulin signaling and synaptic plasticity [61, 62]), and PDE8B (cyclic nucleotide signaling in motor coordination [63]) as well as the levels of key proteins using western blot (Fig. 3d): CNN1 (actin-binding proteins), TPM4 (actin-binding proteins), TRAM-1 (membrane translocation proteins), CAMK2B (synaptic plasticity, Ca^2+^ signaling [61, 62]), and GOPC (ion transport). Despite a previous study demonstrating reduced activity in the Wnt signaling axis of MDS patients [19], we observed an increase in WNT16 in MDS cells (Fig. 3c). This increase could potentially be attributed to increased secretion of Wnt ligands, compensating for the dysregulated Wnt signaling in the MDS condition [64]. More interestingly, CAMK2B (calcium/calmodulin dependent protein kinase II beta) showed a 2.5-fold elevated expression in MDS cells at the protein level (Fig. 3d). CAMK2 is a multifunctional serine/threonine-protein kinase encoding four different isoforms (CaMK2α, CaMK2β, CaMK2γ, or CaMK2δ) involved in neuronal regulation, membrane polarization, learning abilities, and apoptosis of cancer cells [65–67]. Importantly, CAMK2 modulates synaptic plasticity and neurotransmission in response to calcium signaling as a result of neuronal activity [68]. The α- and β-isoforms of CAMK2 are enriched in the brain, activating the Ca^2+^ signaling cascade [61, 62]. One of the common phenotypic features observed in MDS patients is hypotonia due to muscle weakness, which causes elevated calcium levels. Because our transcriptomics analysis identified elevated Ca^2+^ and cAMP-mediated signaling in MDS (Fig. 2c) and CAMK2B was 2.5-fold higher in MDS cells at the protein level (Fig. 3d), we performed a cAMP direct immunoassay, which indirectly measures the calcium levels via cAMP levels. MDS cells exhibited relatively higher cAMP levels than non-MDS cells (Fig. 3e), supporting our pathway analysis and the clinical findings.

MDS patients experience a dysfunctional nervous system; therefore, we probed DEGs that function in synaptic processes and neuronal development. At the RNA level, several notable DEGs were validated using RT-qPCR: BEX1 (axonal regeneration [69]), CDH2 (neuronal migration and axon pathfinding [70]), DLG1 (remodeling of the synapses [71]), GRIA1 (neurotransmitter receptors [72]), KCNN2 (neuronal excitability [73]), NRXN3 (synaptic transmission [74]), SNCA (synaptic vesicle trafficking [75]), SEMA4D (neuron projection development [76]), ST8SIA2 (synaptic plasticity and neural network formation [77]), STX1A (synaptic vesicle exocytosis [78]), and SYT7 (presynaptic function regulator [79]) (Fig. 3f). Similar findings were obtained at the protein level: AHNAK (blood–brain barrier formation, cardiac calcium channel regulation [80]), ARSA (axonal transport [81]), and CAMK2B (synaptic plasticity) (Fig. 3g). In summary, this DEG analysis identified a multitude of targets for developing improved therapeutics for treating MDS patients. Ultimately, these findings should be integrated with transcriptomics and proteomics results from multiple MDS patients and various cell types to capture the entire molecular landscape.

### Overexpression of METTL16 Increases Global Protein Translation in MDS Cells

**Fig. 4:**
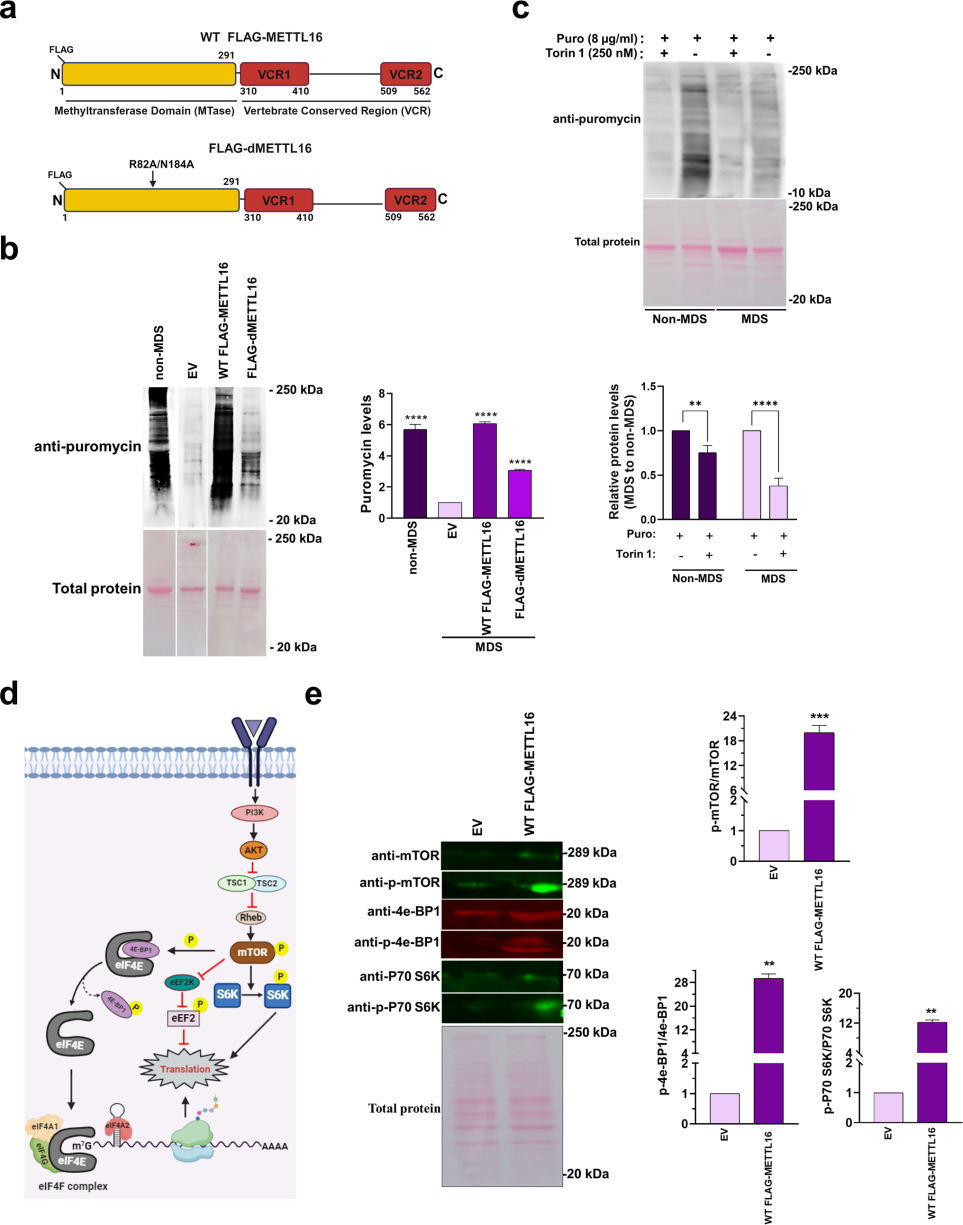
Role of METTL16 in protein translation. **a** Schematics of WT and a catalytically inactive mutant of METTL16 (R82A/N184A). The N-terminal methyltransferase domain is in yellow, and the C-terminal vertebrate-conserved regions (VCR) 1 and 2 are in red. **b** SUnSET assay showing differences in global translation for non-MDS cells and MDS cells transfected with EV, WT FLAG-METTL16, or FLAG-dMETTL16. Puromycin incorporation was normalized using Ponceau S-staining of total protein and then relative to non-MDS sample set an arbitrary value of 1. **c** The dependency on the mTOR pathway was investigated by treating cells with Torin 1 (250 nM), an inhibitor of the mTOR pathway, followed by puromycin treatment to visualize changes in protein translation. Individual non-MDS and MDS values were normalized to the relevant total protein lanes, and then untreated and Torin-treated samples were normalized by setting untreated at an arbitrary value of 1. **d** Schematic showing how the mTOR pathway regulates protein translation; schematic was prepared using BioRender. **e** MDS cells transfected with EV or WT FLAG-METTL16 were quantified for the levels of the mTOR, p-mTOR, and the downstream effectors, 4e-BP1, p-4e-BP1, P70 S6K, and p-P70 S6K in western blot. Total protein was used for the normalizations. All quantitative values are the average ± standard deviation of three biological triplicates (*n* = 3), and *p* values were calculated using a two-tailed unpaired Student’s *t* test. ****p value < 0.0001, ***p value < 0.0002, **p value < 0.0021, *p value < 0.033, ^ns^*p* value < 0.1234

Our proteomics analysis revealed a dramatic downregulation of EIF2 signaling (Fig. 2f), which suggests that global protein levels should be lower in MDS cells than non-MDS cells. METTL16 is a gene encoded in the MDS locus (Fig. 1a); its protein levels are reduced by 50% in MDS cells (Fig. S5) and has been implicated in translation. Although METTL16 is an m^6^A (*N*^6^-methyladenosine) RNA methyltransferase that methylates RNAs in the nucleus, it promotes translation independent of its methyltransferase activity [82–85]. First, we examined the subcellular localization of METTL16 via subcellular fractionation followed by western blot. As observed in other cell lines [86], METTL16 is predominantly localized in the cytoplasm of both non-MDS and MDS cells (Fig. S9). Because cytoplasmic METTL16 mediates protein translation, our next objective was to quantify global protein levels and to determine the relationship between METTL16 and global protein levels. Therefore, we performed a surface sensing of translation (SUnSET) assay. When less than 10 µg/ml puromycin (puro) is used in the SUnSET assay, the incorporation of puromycin directly reflects the integration of the neosynthesized proteins [87, 88]. We confirmed the assay was working by treating cells with 100 µg/ml cycloheximide (CHX), a translational inhibitor, along with puromycin (8 µg/ml) and observed reduced global protein levels in MDS cells compared to non-MDS cells (Fig. S10). Next, we overexpressed two different N-terminal FLAG-tagged versions of METTL16 in MDS cells: WT (FLAG-METTL16) and a methyltransferase-inactive form (FLAG-dMETTL16) (Fig. 4a). Western blot analysis showed that overexpression of FLAG-tagged METTL16 in MDS cells was at a similar level as METTL16 in non-MDS cells (Fig. S11). Non-MDS and MDS cells expressing WT FLAG-METTL16 showed similar levels of protein synthesis while MDS cells expressing the empty vector (EV) or FLAG-dMETTL16 exhibited reduced protein translation (Fig. 4b). Because METTL16 regulates SAM homeostasis, these findings suggest that global protein levels may be interconnected with the mTOR pathway and/or methionine cycle.

Previous studies have shown mTOR signaling as one of the key regulators and an activator of protein synthesis [89, 90]. To determine if the translational effects depend on mTOR signaling, we treated non-MDS and MDS cells with Torin 1, a competitive inhibitor of the mTOR pathway [91]. The SUnSET assay showed decreased translation when non-MDS and MDS cells were treated with Torin 1, suggesting that the mTOR pathway contributes to differences in protein translation (Fig. 4c). mTOR activation (Fig. 4d) is mediated through the downstream phosphorylation of its major effectors: S6K or P70 S6K (ribosomal protein S6 kinase) and 4e-BP1(eukaryotic initiation factor 4E-binding protein) [92]. S6K is activated via direct phosphorylation of Thr389 by p-mTOR, which then subsequently turns on the translation machinery to initiate translation [93]. Similarly, unphosphorylated 4e-BP1 binds to eIF4E, preventing the eIF4F complex assembly. Upon phosphorylation of Thr37/46 by p-mTOR, 4e-BP1 dissociates from eIF4E, and translation initiation can proceed [94]. Therefore, we examined the phosphorylation states of S6K, mTOR, and 4e-BP1 in MDS transfected with either the empty vector or WT FLAG-METTL16. Importantly, phosphorylation was enhanced when WT FLAG-METTL16 was overexpressed (Fig. 4e). We conclude that higher levels of METTL16 increase global protein translation in an mTOR-dependent manner via increased phosphorylation of S6K, mTOR, and 4e-BP1.

### Cellular Methylation Potential Is Reduced in MDS Cells

**Fig. 5:**
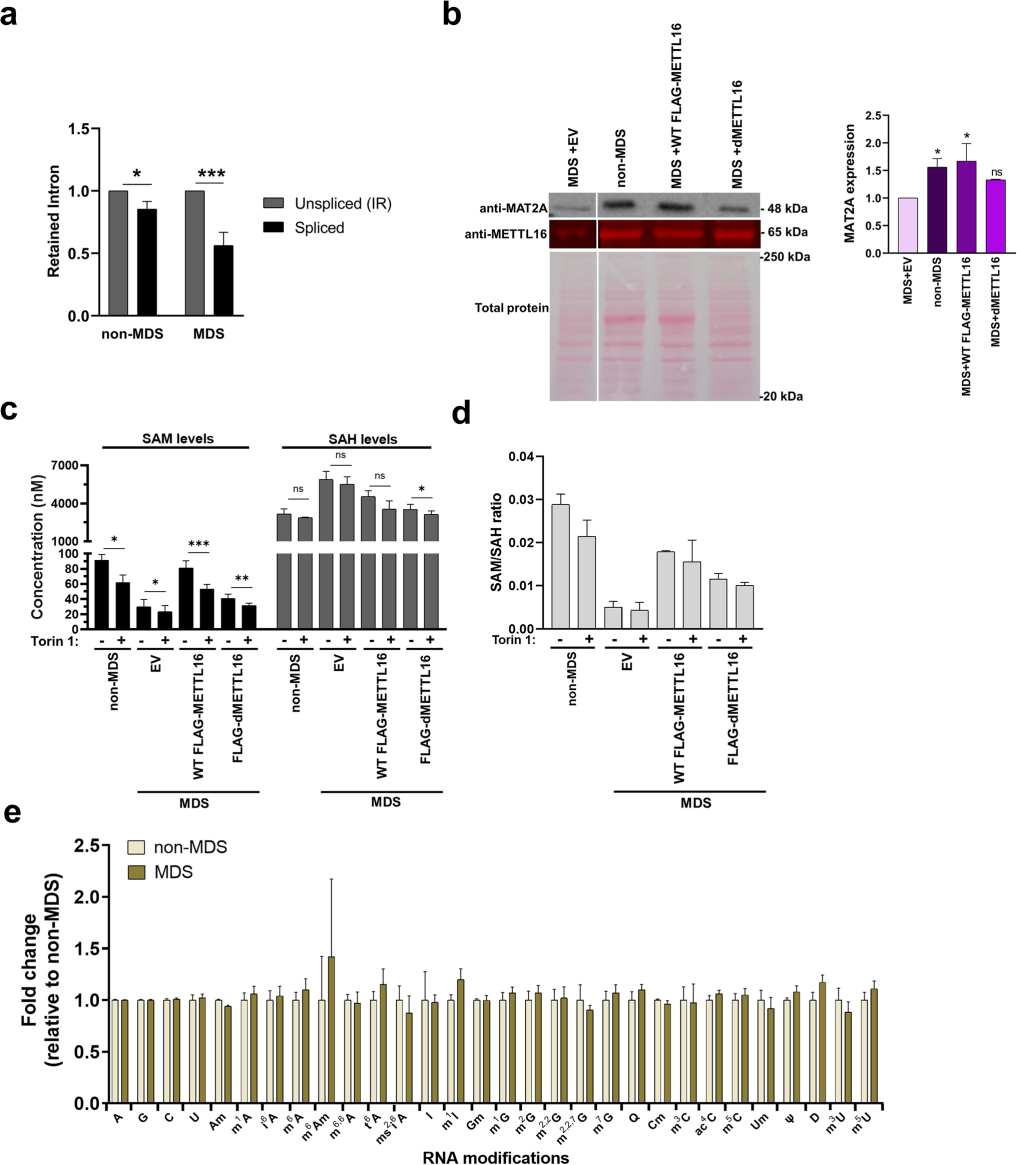
MAT2A levels and SAM homeostasis is perturbed in MDS cells. **a** RT-qPCR quantitation of MAT2A isoforms: unspliced intron-retained (gray) and spliced (black). Each isoform was normalized to GAPDH, and then unspliced was set at an arbitrary value of 1 for each cell line. **b** MAT2A protein expression in non-MDS cells versus MDS cells transfected with empty vector (EV), FLAG-METTL16, or FLAG-dMETTL16. MAT2A levels were normalized to total protein and then normalized to the EV sample, which was set at an arbitrary value of 1. **c** SAM and SAH levels were measured using a SAM/SAH ELISA combo kit for untreated ( −) and Torin 1-treated ( +) cells. These values were then used to calculate the **d** SAM/SAH ratio (or methylation index). **e** Unmodified and modified RNA nucleoside abundance was determined using UPLC-MS/MS. All quantitative values are the average ± standard deviation of five biological replicates, and *p* values were calculated using a two-tailed unpaired Student’s *t* test. Please note that RNA nucleoside levels did not show statistically significant fold changes. ****p value < 0.0001, **p value < 0.0021, ^ns^*p* value < 0.1234 using two-tailed unpaired Student’s *t* test

mTOR promotes the production of SAM by regulating the expression of MAT2A (Fig. S12) [95]. METTL16 regulates SAM levels via methylation, and splicing of MAT2A mRNA and SAM levels can regulate mTOR signaling via the SAMTOR-GATOR complex, which senses SAM to enhance mTOR signaling [96]. First, we employed RT-qPCR to determine if there was a difference in spliced versus the intron-retained isoform of MAT2A mRNA, and indeed MDS cells have a smaller fraction of spliced MAT2A mRNA (Fig. 5a). Western blot analysis confirmed MAT2A is expressed 1.5-fold greater in non-MDS than MDS cells (Fig. 5b). Furthermore, overexpression of WT FLAG-METTL16, but not FLAG-dMETTL16, in MDS cells restored MAT2A levels to that of non-MDS cells (Fig. 5b). In addition to altered MAT2A levels, our proteomics results showed that the downstream enzyme responsible for the conversion of homocysteine to cystathionine (i.e., cystathionine beta synthase or CBS, which catalyzes the first committed step of transsulfuration pathway) was downregulated by 1.8-fold in MDS cells (Supplementary file 2) and confirmed via western blot (Fig. 3b) [97]. Collectively, these findings suggest that intracellular SAM levels may be different in non-MDS versus MDS cells. Using a SAM/SAH ELISA combo kit, SAM levels were 3-fold lower, and SAH levels were twofold higher in MDS cells (Fig. 5c), leading to altered methylation potential of eightfold (Fig. 5d). The methylation potential was partially restored by 55% and 40% upon overexpression of WT FLAG-METTL16 and FLAG-dMETTL16, respectively (Fig. 5d). This result suggests that the levels of METTL16 and its catalytic activity contribute to SAM homeostasis (Fig. 5c–d). To confirm SAM homeostasis depends on the mTOR pathway, we measured the levels of SAM and SAH in the presence of Torin 1. Torin 1-treated samples had lower SAM levels and higher SAH levels than untreated samples, supporting that SAM regulation by METTL16 occurs via the mTOR pathway (Fig. 5c–d). These results suggest that the cellular state of MDS cells may be improved via restoration of SAM levels.

These changes in SAM/SAH levels could possibly impact SAM-dependent methylation events targeting DNA, RNA, and proteins. Therefore, we examined the global levels of 33 RNA nucleoside modifications (mostly methyl-containing modifications, see Table S5) along with the canonical (A, G, C, U) ribonucleosides using UPLC-MS/MS. For the 26 modifications that were detected (Supplementary file 5), the relative fold change values were calculated with the non-MDS being normalized to 1. However, no significant changes in the relative abundance of the 26 detectable RNA nucleoside modifications, including m^6^A, were observed (Fig. 5e). Although global levels may be unchanged, it is possible that key site-specific modifications, such as hairpin 1 of MAT2A mRNA, could be perturbed because global RNA modifications will be dominated by signals from highly abundant rRNAs and tRNAs [82]. Lastly, it is also important to note that the methionine cycle involving SAM is linked to redox homeostasis through the glutathione cycle, and glutathione influences the glutamatergic neurotransmission system [98]. However, our pathway analyses of DEGs at the RNA and protein levels did not identify any hits (Supplementary files 3 and 4).

### Overexpression of METTL16 Restores Cell Migration

**Fig. 6:**
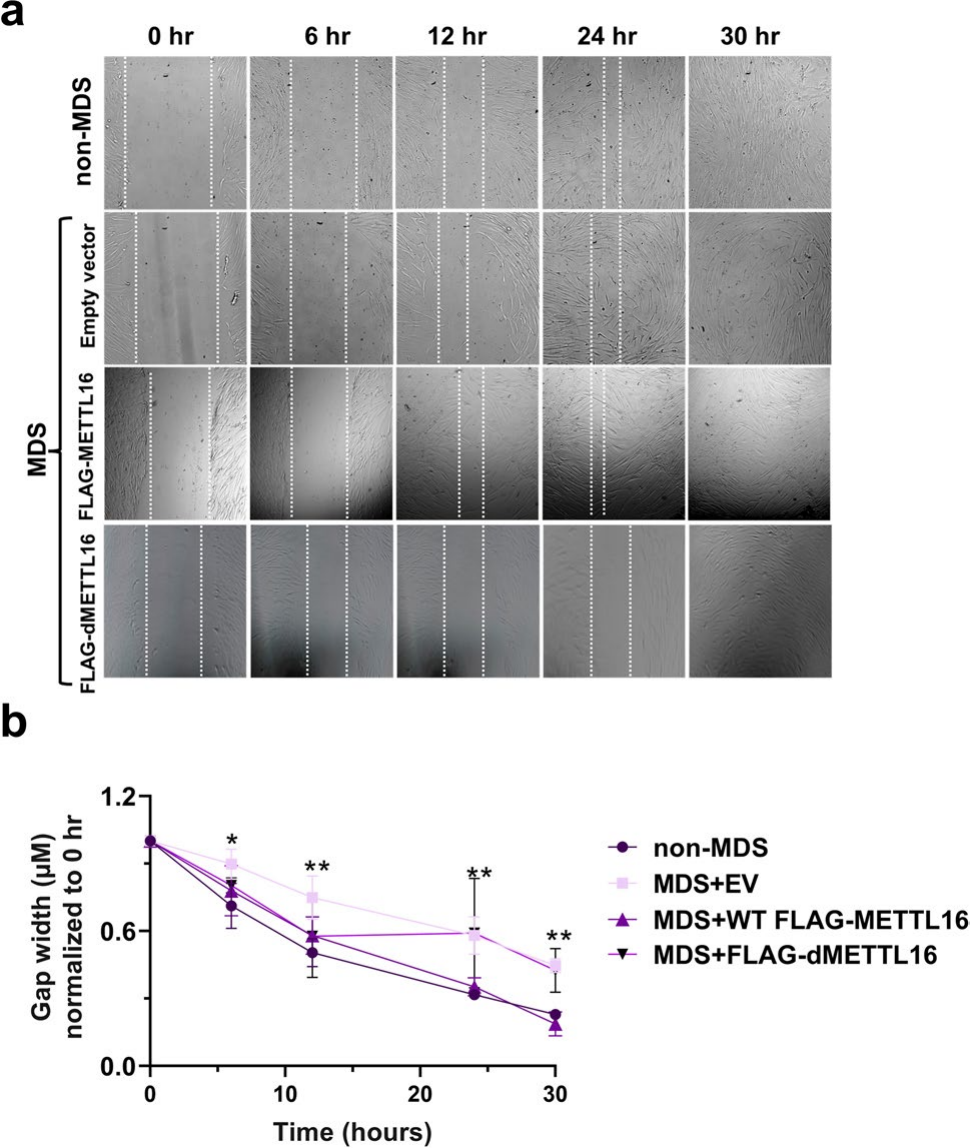
Overexpression of METTL16 rescues cell migration defect in MDS cells. **a** Representative live-cell images of cells from the wound-healing assay at 0-, 6-, 12-, 24-, and 30-h time points when WT FLAG-METTL16 and FLAG-dMETTL16 are overexpressed in MDS cells. **b** A plot of gap-width measurements is shown. All gap-width measurements were normalized to their respective 0-h measurements. Values represent averages ± standard deviation of three biological triplicates (*n* = 3). Statistical analysis performed using a two-tailed unpaired Student’s *t* test showed no significant changes. **p value < 0.0021, *p value < 0.033 using two-tailed unpaired Student’s *t* test

Defects in neuronal migration are well established and have been linked to the *PAFAH1B1*, *YWAHE*, and *CRK* genes [99]. Because the expression level of METTL16 impacts global protein and SAM/SAH levels, we also wondered if METTL16 contributes to the cell migration defect because (i) it is a gene in the MDS locus (Fig. 1a) whose expression in MDS cells is 50% less than in non-MDS cells at both the RNA and protein levels (Fig. 1e and S5) and (ii) depletion of METTL16 in hepatocellular carcinoma, osteosarcoma, and colorectal cancer cell lines decreases cell proliferation, migration, and invasion [83, 100, 101]. Therefore, we overexpressed WT and the catalytically dead mutant of FLAG-METTL16 (Fig. 4a) in MDS cells to determine if the cell migration defect could be rescued in the context of a wound-healing assay. Gap-width measurements of the wound area in MDS cells overexpressing WT FLAG-METTL16, but not FLAG-dMETTL16, showed relatively smaller gap width than MDS and was similar to non-MDS cells (Fig. 6a–b). Thus, a catalytically active METTL16 is required to restore the migrative defects, suggesting that the haploinsufficiency of METTL16 plays a role in cell migration as does PAFAH1B1, YWAHE, and CRK [99].

## Discussion

As a rare disease, MDS could benefit from more in-depth molecular-level studies [11]. Understanding the gene deletions (both protein-coding and non-protein coding genes) and pathway malfunctions beyond the MDS locus could provide insights into the potential regulators driving the abnormalities and symptoms observed in MDS patients. Like our multi-omics study, some previous studies have utilized control/non-MDS (BJ cells) and MDS (GM06097 cells) [20, 21]. For example, a single-cell RNA-seq study using MDS-patient-derived human iPSC organoids, which included BJ and GM06097 cells as well as additional MDS cell lines, and a CRISPR-knockout study provided molecular insights into (i) cell migration defects resulting from MDS-locus gene deletions such as *PAFAH1B1*, *YWHAE* and (ii) other genes that are regulators of cortical developmental malformation in MDS (*ASPM*, *PAX6*, *SOX2*, *NDEL1*, *CTIP2*) [21, 102]. Similarly, another study of MDS patient-derived organoids established destabilized microtubule organization and impaired β-catenin activation in the N-cadherin/β-catenin signaling axis of MDS, reiterating the importance of PAFAH1B1 and YWHAE in diminished cell migration during cortical development [19]. However, as most of the studies focused on neuronal migration defects, other severe phenotypic features of MDS patients are understudied. In addition to cell migration, our study identified defects in calcium levels, global protein translation, and SAM homeostasis, which collectively may underlie a majority of MDS pathologies in any cell type of an MDS patient because protein translation and intracellular metabolite levels are essential to maintaining a healthy cellular state.

Earlier clinical studies of MDS patients have reported organ malfunctions in the kidney, heart, and liver [41]. A recent autopsy report of a 4-year-old female MDS patient, who had a deletion spanning p13.3 and p13.2 on chromosome 17 similar to the MDS cells used herein (Fig. 1a), presented with multiorgan failure, cardiorespiratory arrest, congested liver, extensive renal autolysis, increased nose and mouth secretions, and typical syndromic facial features of MDS including an upturned short nose [41]. Our multi-omics study highlights specific genes and pathways that are linked to these organ functionalities: cardiac hypertrophy (CACNG4, GJA5, THBS2, FBLN1), skeletal system development (ADD2, TRAM-1, NEAS), calcium signaling (CAMK2B, PDE8B), synaptogenesis (APOE, GABBR2, ARSA, BEX1), and STAT3 pathway (WNT16) (Fig. 2c). The expression of CAMK2B was enhanced at both the RNA (~ 10-fold) and protein levels (~ 2.5-fold) (Fig. 3c–d). CAMK2 signaling engages in Ca^2+^-dependent signaling during early stages of the postnatal and mature brain development [61]. However, the function of CAMK2B in neuronal functioning remains an enigma due to the high redundancy between the two isoforms, CAMK2A and CAMK2B [61]. Because calcium in cardiac muscles is regulated by calcium/calmodulin-dependent protein kinase II (CaMKII) and has a role in cardiac muscle contraction, hypertrophy, and gene expression [103], the observed elevated CAMK2B in MDS appeared to be a major reason for dysregulated calcium signaling. Higher cAMP levels indicate higher Ca^2+^ levels in MDS cells (Fig. 3e), reinforcing the molecular link between heart development and altered calcium signaling. For STAT3 signaling, STAT3 activation leads to enhanced ischemic neuronal damage upon upstream IL (interleukins) binding to the receptors, and STAT3 suppression has the ability to restore the neurological defects [104, 105]. Our transcriptomics pathway analyses illustrated an activated STAT3 pathway and decreased IL-1β signaling in MDS (Fig. 2c and Fig. S3). The STAT3 pathway is often targeted for potential drug therapeutics for neurodegenerative disorders. For example, STAT3 phosphorylation and neuronal autophagy are promoted by brain-derived neurotrophic factor (BDNF), a promising agent for targeting Parkinson’s disease [106]. Another example shows that inhibiting STAT3 during brain inflammation prevents astrogliogenesis and aids in neurogenesis [43]. Therefore, pursuing the possible downstream regulators of JAK/STAT pathway in detail could be a plausible starting point in designing better therapeutics against MDS. The FDA-approved Janus kinase inhibitors (JAKi) (i.e., jakinibs) are used to diminish the effects from STAT3 activation and to control progression of neurodegenerative diseases such as Alzheimer’s and Parkinson’s diseases [107, 108]. Baricitinib, another FDA-approved JAKi, blocks interferon responses in Alzheimer’s disease [109] while the JAK2 inhibitor AZD1480 lessens neuroinflammation and neurodegeneration in Parkinson’s disease [110]. Similarly, an alternative therapeutic approach to JAKi would be the use of NGF and EGF treatments to enhance protein translation pathways in MDS because global protein levels are lower in MDS cells (Fig. 4b–c) [47].

Our multi-omics study has revealed that another MDS-locus gene (Fig. 1a), *METTL16*, plays a pivotal role in MDS. The mRNA and protein levels of METTL16 were reduced by 2-fold (Fig. 1e, S5, and S11), contributing to decreased protein translation (Fig. 4b), lower methylation potential (Fig. 5d), and poor cell migration (Fig. 6) in MDS cells. mTOR signaling is known to regulate all of these processes: translation, metabolism, and cell growth [111]. This intricate regulation of SAM by METTL16 and its interaction with the mTOR pathway reveals a new therapeutic opportunity. The disruption of mTOR signaling by METTL16 may contribute to any MDS symptom but most especially severe neurological abnormalities [14] that cause intellectual disabilities, seizures, and epilepsy [15], congenital heart abnormalities (enhanced hypertrophy, ventricular septal defect) [16], gastrointestinal and/or kidney functionalities (multicystic dysplastic kidney), and motor coordination impairments [10]. Thus, careful activation of mTOR signaling may be clinically beneficial for MDS patients. Although there are FDA-approved mTOR inhibitors, there are no mTOR activators, although some are being developed [112, 113]. Another therapeutic option is to restore the imbalance in SAM/SAH levels. MDS cells have a low SAM/SAH ratio (Fig. 5c–d), and CBS, an enzyme in the methionine cycle that utilizes homocysteine in the transsulfuration pathway, is downregulated (Fig. 3b). The diminished SAM/SAH ratio in MDS cells could contribute to two phenotypes of MDS patients. As enhanced homocysteine levels (homocystinuria) lead to ischemic heart injuries [114], this would be consistent with the cardiac defects observed in MDS patients (Fig. 2c). Separately, as homocysteine is an agonist at the glutamate-binding site of the *N*-methyl-d-aspartate receptors in the brain/neurons, elevated levels of homocysteine could contribute to neurotoxicity [115]. Therefore, the low SAM/SAH ratio in MDS patients may contribute to the cardiac and neurological defects identified from our pathway studies (Fig. 2c). Similarly, our GO analyses showed enhanced NE secretion and transport (Fig. 2b). Because PNMT (phenylethanolamine-N-methyl transferase) is a SAM-dependent enzyme that converts norepinephrine to epinephrine, the low SAM levels in MDS cells may partly contribute to increased NE secretion and result in congestive heart failures [116]. Therefore, we speculate that the enhanced NE secretion in MDS cells could potentially be connected to the cardiac hypertrophy condition observed in MDS patients. Collectively, restoring SAM homeostasis could be beneficial for MDS patients. Administration of betaine (an FDA-approved alternative methylating agent) reduces elevated SAH levels as a means to restore SAM-dependent methyltransferases activity [117], improves methylation of homocysteine to limit cardiovascular diseases (i.e., atherosclerosis) [118], and shows anti-epileptic properties by blocking homocysteine-induced seizures in rats [119, 120]. Thus, betaine may be a plausible therapeutic for multiple symptoms observed in MDS patients. Additionally, targeting gene candidates interrelated to neuronal-linked pathways (synaptogenesis, neuronal action potential), cell signaling pathways (Wnt signaling, Rho-GTPase signaling, and EIF2 signaling in protein translation), and cytoskeleton formation (cell adhesion molecules, ion channel complexes) represent new opportunities to develop therapeutics for MDS patients. Our pathway analyses confirmed altered Wnt signaling in MDS cells (Fig. 3c and S2c), supporting previous studies showing how alterations in the organization of the cell microtubule network in vertical radial glial of MDS organoids lead to disturbed Wnt/β-catenin signaling axis [19].

MDS cells exhibited an 8% decrease in viability, a longer doubling time, and a 15% decrease in cell proliferation (Fig. 1b–d). GM06047 cells grew even slower. Because most MDS patients die in utero, we speculate that decreased cell viability and slower cell growth contribute directly to MDS and could be an underlying cause for a multitude of symptoms: heart abnormalities (enhanced hypertrophy, large ventricles, ventricular septal defect) [16], scoliosis (sideways curve of the spine) [121], gastrointestinal and/or kidney functionalities (multicystic dysplastic kidney) [10], and neuronal abnormalities (seizures) [15]. METTL16 is essential for mouse embryonic development and critical for human cellular differentiation, suggesting it may have a larger role in MDS than currently appreciated [122–125]. METTL16 having a role in cell migration may partially explain why PAFAH1B1 haploinsufficiency alone did not recapitulate the MDS severities reported [21]. PAFAH1B1 truncations affect the cytoplasmic dynein complex in microtubule-based transport and neuronal migration [126, 127], and the METTL16 counterpart, METT-10, in *Caenorhabditis elegans* interacts with the dynein light chain-1 (DLC-1) to inhibit cell proliferation [128]. We examined published human METTL16 co-immunoprecipitation studies but none of the protein-binding partners suggest a connection to cell migration [129]. However, these co-immunoprecipitation studies have identified protein-binding partners involved in translation (e.g., eIF3a, eIF3b [83], and eIF4E2 [84]), which may mechanistically explain why the decrease of METTL16 contributes to less protein synthesis (Fig. 4b–c). Unfortunately, the global levels of nucleoside modifications did not yield any significant changes among non-MDS and MDS cells (Fig. 5e). Potential reasons could be relatively lower levels of mRNA for detection, as this study was conducted with total RNA extracted from non-MDS and MDS cells, and METTL16 is known to methylate small nuclear RNAs and mRNAs so far. However, a comparative analysis of site-specific modifications may pinpoint key changes in m^6^A or other modifications sensitive to SAM/SAH levels [82, 122, 130–132]. This comparative analysis may be insightful because m^6^A is the most abundant mRNA modification in the brain and is involved in neurogenesis, memory, and brain volume [133]. These findings highlight METTL16 as one of the candidate genes to further investigate in MDS. In addition, this study captures DEGs for only a single MDS patient and cell type. Gene deletions and other chromosomal variations (e.g., ring formation) are inherent to MDS, leading to different clinical presentations. One such example was observed in this study: noticeably slower growth for GM06047 cells, which have a ring chromosome. Thus, more MDS patient samples need to be examined to identify which genes and pathways are commonly affected and to establish clear genotype–phenotype relationships for patient-specific therapeutic interventions.

Given the high mortality rate of MDS patients in utero and the limitations of cell culture models in fully capturing how embryonic development impacts the disease, exploring an in vitro mouse study could be valuable for modeling MDS. Such a study would provide insights into the influence of embryonic development on MDS. However, it is important to note that the mouse brain is naturally lissencephalic and may not fully replicate certain aspects of cortical development. Therefore, utilizing skin fibroblasts from healthy and MDS-affected infants, as they closely resemble early brain development [134], would offer a more relevant platform for studying MDS at the molecular level, considering their biochemical and metabolic similarities to neurons. Taken together, our study highlights the possible involvement of genes within (i.e., METTL16) and beyond the MDS locus and their relevant contributions to the phenotypic features. Notably, CACNG4 (enhanced cardiac hypertrophy), ADD2 (low muscle tone), SPTAN1 (epilepsy), SHANK2 (retinal defects), WNT (neuronal abnormality), GABBR2 (neuronal abnormality), CAMK2B (enhanced cardiac hypertrophy/neuronal defects), BEX1 (neuronal defects), and ARSA (neuronal defects) are genes exhibiting significant expression changes in MDS cells at the RNA and protein levels. Our multi-omics results support therapeutic strategies that suppress the STAT3 pathway (e.g., JAKi), enhance protein translation (e.g., NGF and EGF treatments), activate the mTOR pathway, and restore the SAM/SAH methylation index (e.g., betaine treatment) as a means to improve multiple symptoms of MDS patients. Hence, our work provides a more comprehensive platform for establishing direct links between genes and the phenotypes of MDS patients and for identifying drug targets for MDS.

## Supplementary Information

Below is the link to the electronic supplementary material.

Supplementary file1 (XLSX 204 KB)

Supplementary file2 (XLSX 429 KB)

Supplementary file3 (XLSX 2.30 MB)

Supplementary file4 (XLSX 147 KB)

Supplementary file5 (XLSX 12.9 KB)

Supplementary Information (PDF 3.66 MB)

## Data Availability

RNA-seq data have been deposited in NCBI’s Gene Expression Omnibus (GEO) under accession number GSE247527. All other data are provided within the manuscript or as supplementary files (.xlsx), which include results from RNA-seq, mass spectrometry, and pathway analyses.
