# Supplementary material for: Multi-Omics Approach Reveals Genes and Pathways Affected in Miller-Dieker Syndrome
Source: Mol Neurobiol. 2024 Nov 7;62(4):5073–94. doi: 10.1007/s12035-024-04532-7 (PMC11880102; doi:10.1007/s12035-024-04532-7)
Supplement: Supplementary file 6 — Supplementary Information (PDF 3.66 MB) [file 12035_2024_4532_MOESM6_ESM.pdf]

SUPPLEMENTARY INFORMATION

Multi-omics approach reveals genes and pathways affected in Miller-Dieker Syndrome

Gowthami Mahendran[1], Kurtis Breger[1], Phillip J. McCown[1,‡], Jacob P. Hulewicz[1], Tulsi Bhandari[2], Balasubrahmanyam Addepalli[2], Jessica A. Brown[1,*]

[1]Department of Chemistry and Biochemistry, University of Notre Dame, Notre Dame, IN 46556, USA
[2]Department of Chemistry, University of Cincinnati, Cincinnati, OH 45221, USA

[‡]Current address: Michigan Medicine, Department of Internal Medicine, Division of Nephrology, University of Michigan, Ann Arbor, Michigan 48109, USA

* To whom correspondence should be addressed:
Tel: 1 (574) 631-6486
Email: jbrown33@nd.edu

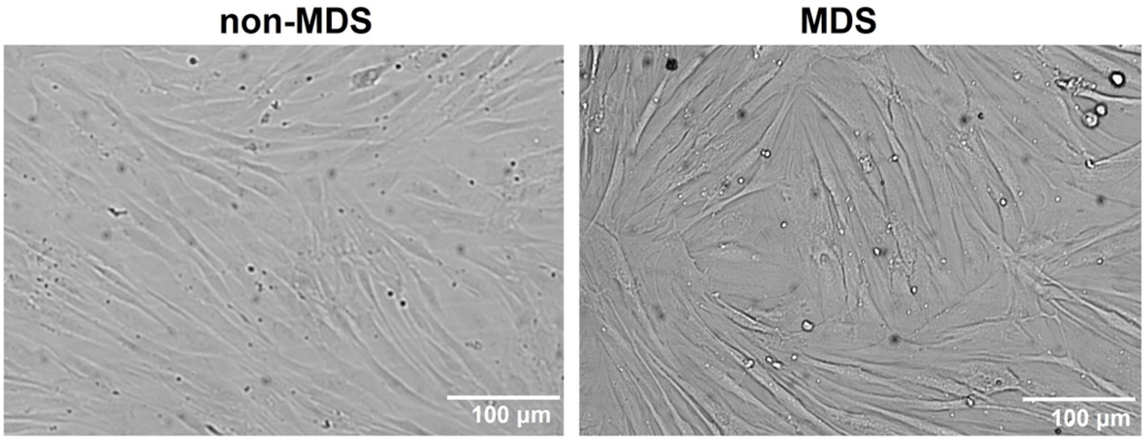

Fig. S1 Morphological features of BJ (non-MDS) and GM06097 (MDS) cells. Images were acquired on the fifth day after seeding non-MDS (left) and MDS (right) cells. Representative images were captured using a 20× objective on a TE2000 inverted microscope (Nikon) with a Hamamatsu CMOS camera connected to NIS-Elements BR 413.04 64-bit software (Nikon). Scale bars represent 100 µm

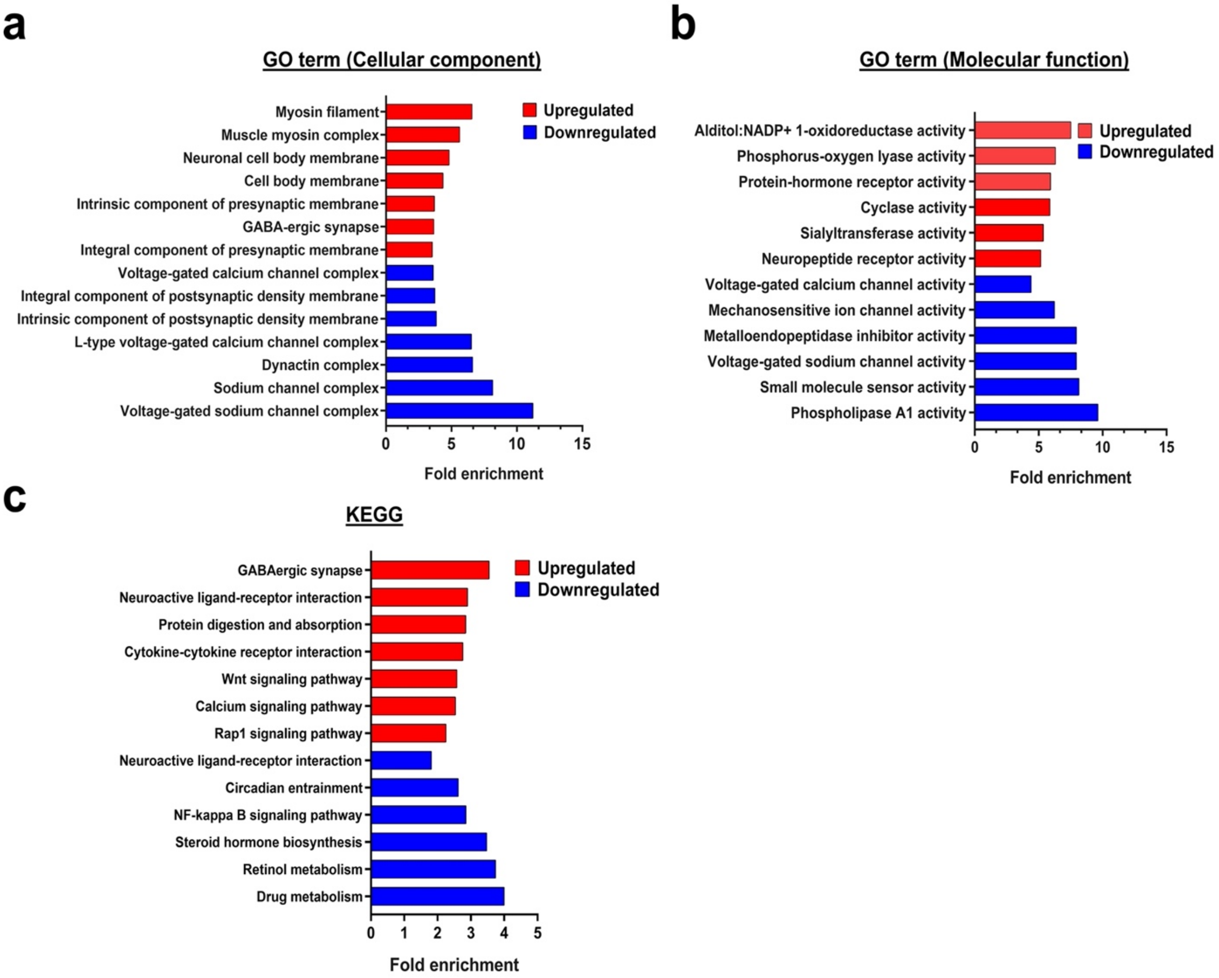

Fig. S2 Pathway analysis of the RNA-seq results using Shiny GO 0.76.3 Gene ontology terms of **(a)** cellular component (CC) and **(b)** molecular functions (MF) to determine enriched genes at the RNA level. **(c)** Fold enrichment is shown for DEGs analyzed using KEGG pathway tool in Shiny GO

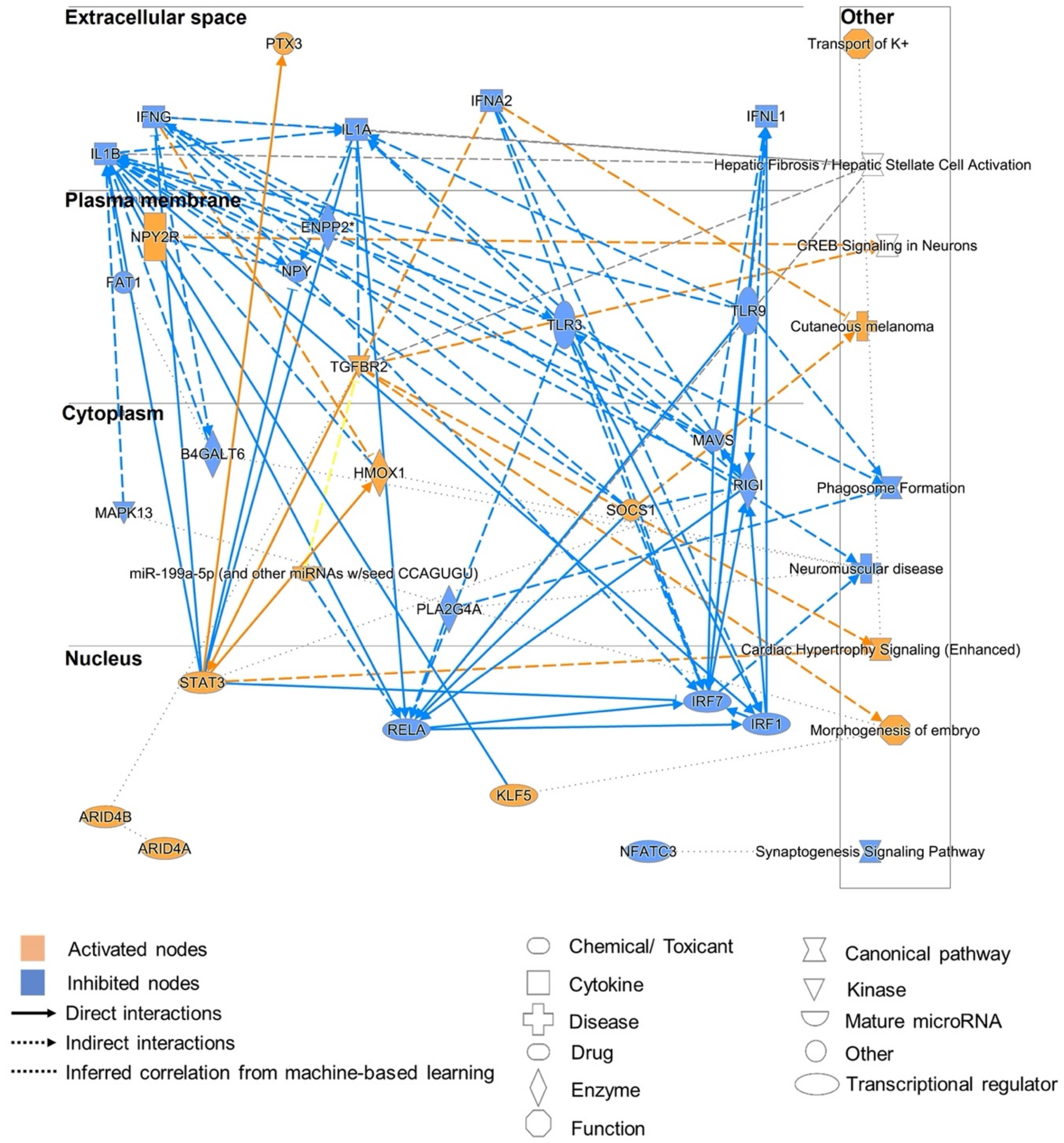

Fig. S3 QIAGEN IPA of RNA-seq results. This graphical summary shows connections among major biological themes (CREB signaling, cardiac hypertrophy, neuromuscular diseases, transport of K^+ etc.), associated pathway components, upstream regulators, and associated diseases. Activated nodes (orange) have a positive z-score ≥2 and inhibited nodes (blue) have a negative z-score ≤ -2. At the bottom, there is a legend to define network and pathway shapes. Solid lines indicate direct interactions, and dashed lines indicate indirect or inferred interactions. Adapted from Qiagen IPA website (https://qiagen.my.salesforce-sites.com/KnowledgeBase/articles/Knowledge/Legend)

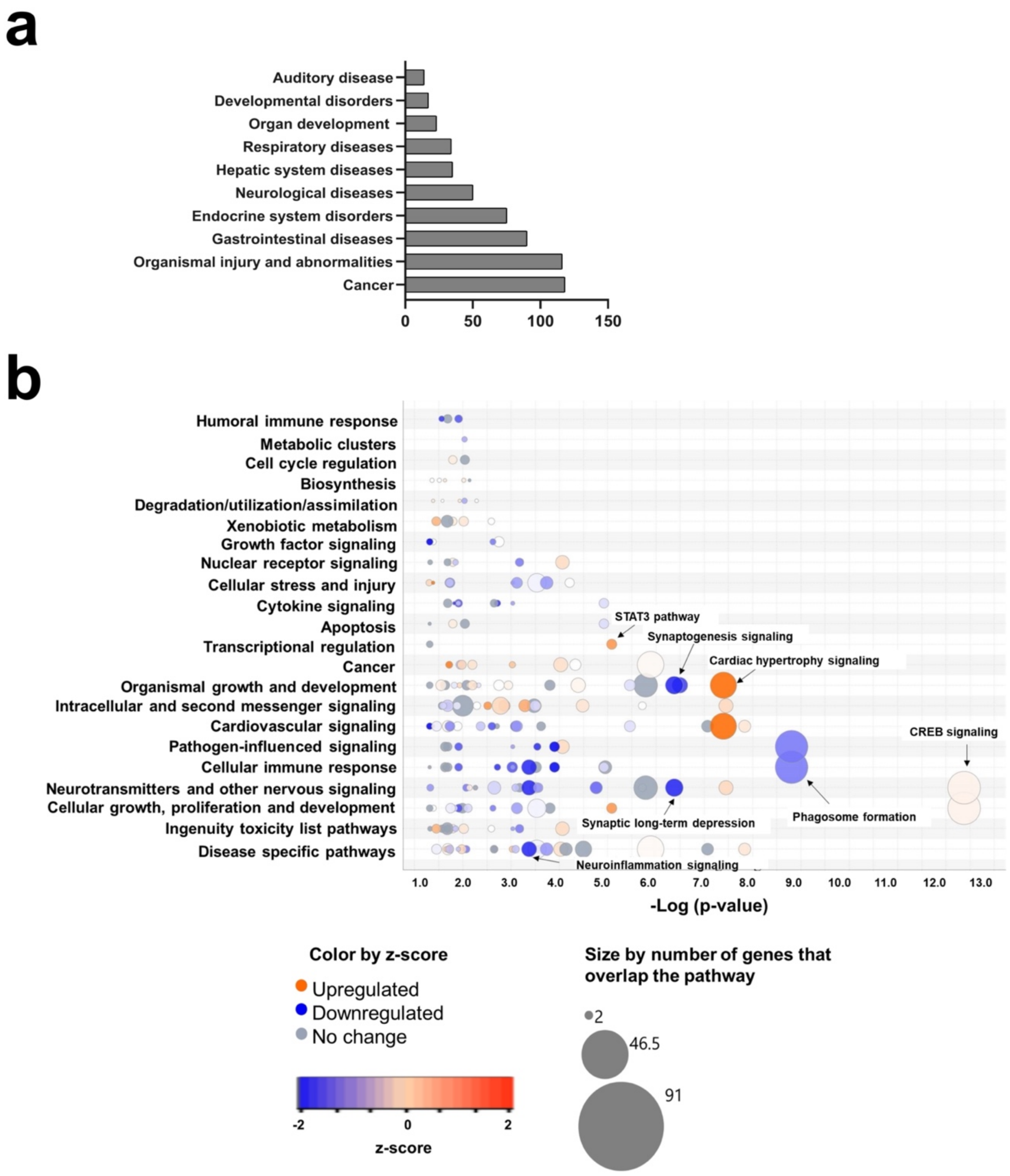

Fig. S4 Qiagen IPA disease and function analysis of RNA-seq results. (a) The bar graph depicts relevant biological processes and diseases predicted to have significant alterations in MDS according to ingenuity ontology. IPA calculates p-values using Fischer's exact test p-value cut-off of 0.05 and range of 1.4 < $\log_2$ fold change > -1.4. **(b)** Bubble chart depicting multiple pathway categories associated with top canonical pathways (see **Fig. 2c**) having significant -log (p-value) (cardiac hypertrophy signaling, STAT3 pathway, synaptogenesis signaling etc.). The size of the bubble corresponds to the number of genes involved in the overlapping pathways. Activated nodes with a positive z-score ≥2 is orange, inhibited nodes with a negative z-score ≤ -2 are blue and pathways lacking a significant activity pattern are gray

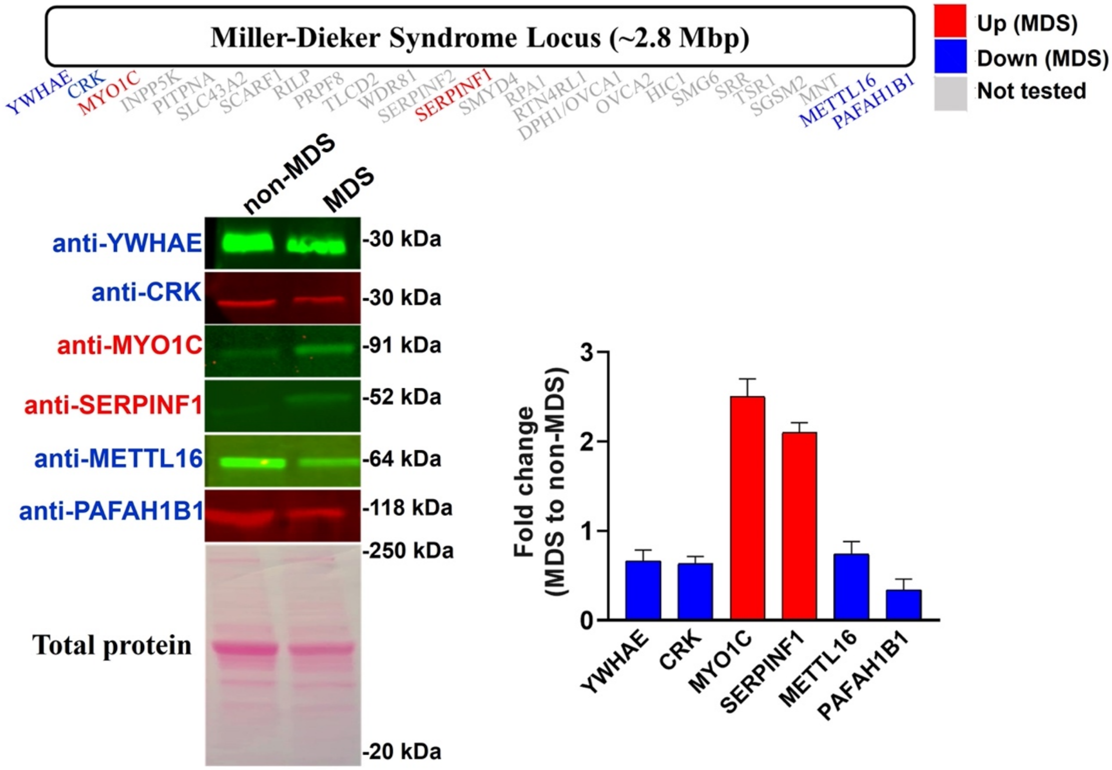

Fig. S5 Protein expression levels of genes encoded in MDS locus. Selected MDS locus genes from MS/MS results were validated using western blot. Four genes were downregulated (blue) and 2 genes were upregulated (red). Representative western blots probed for relevant antibodies are shown next to the respective bar plot. Representative western blots are next to the respective bar graph, quantifying the fold change of protein expression of MDS relative to non-MDS cells after total protein normalization. Fold change values are an average ± standard deviation of biological triplicates (n=3)

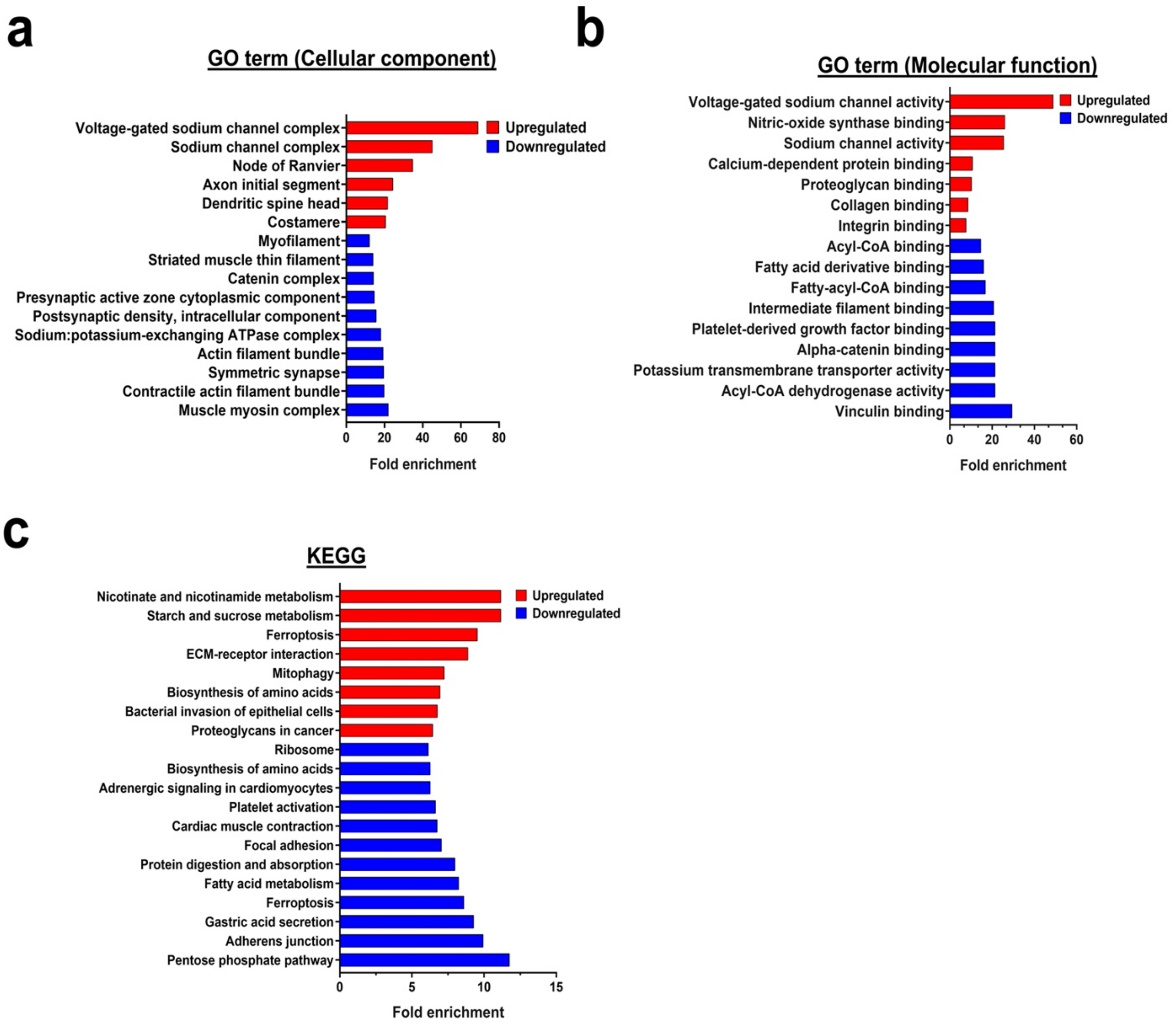

Fig. S6 Pathway analysis of the proteomics results using Shiny GO 0.76.3 Gene ontology terms of **(a)** cellular component (CC) and **(b)** molecular functions (MF) to determine enriched genes at the protein level. **(c)** Fold enrichment is shown for DEGs analyzed using KEGG pathway tool in Shiny GO

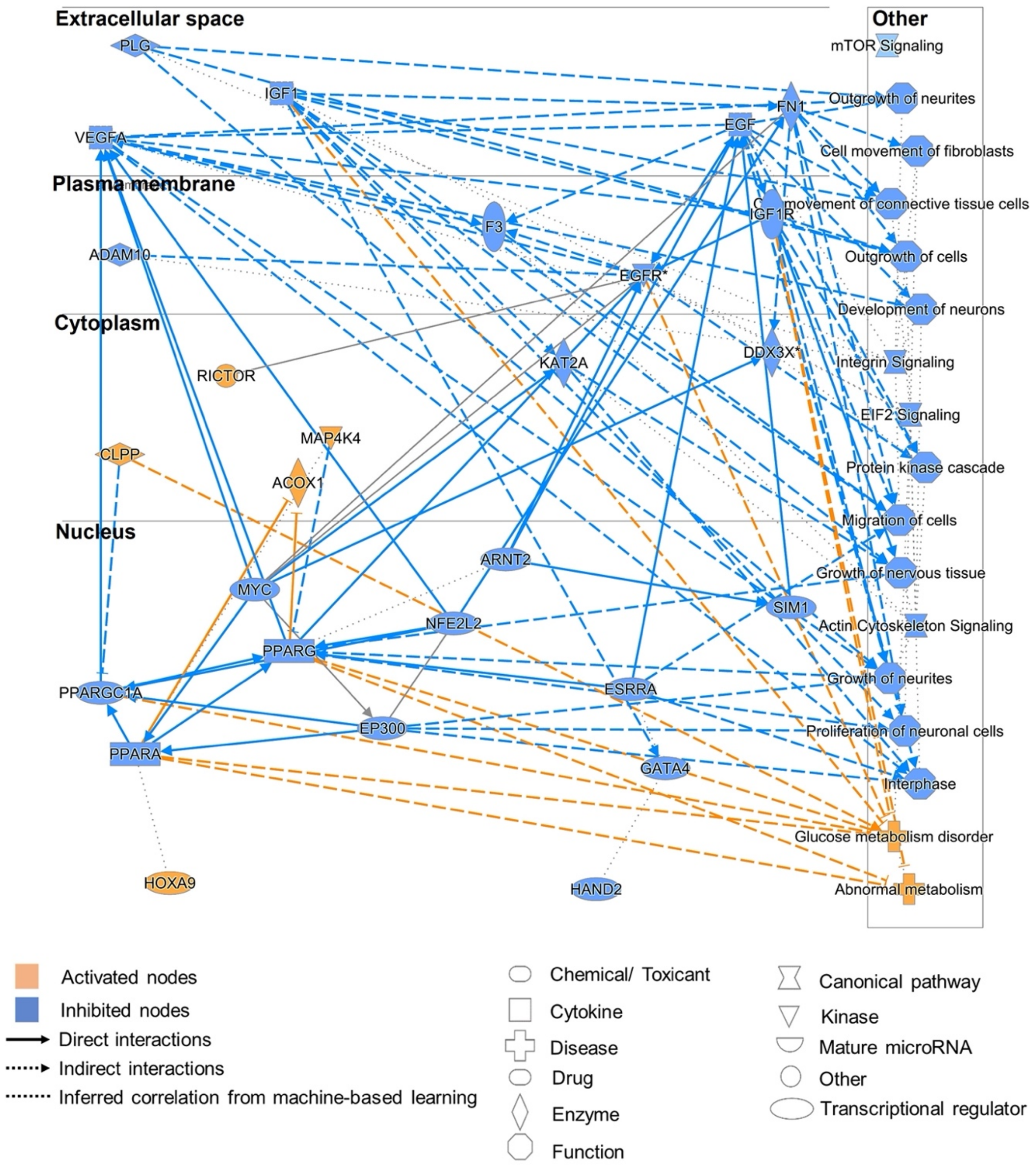

Fig. S7 QIAGEN IPA Analysis of proteomics results. This graphical summary shows connections among major biological themes (EIF2 signaling, actin cytoskeleton signaling, integrin signaling, development of neurons etc.,), associated pathway components, upstream regulators, and associated diseases. Activated nodes (orange) with a positive z-score ≥2 and inhibited nodes (blue) with a negative z-score ≤ -2. At the bottom, there is a legend to define network and pathway shapes. Solid lines indicate direct interactions, and dashed lines indicate indirect or inferred interactions. Adapted from Qiagen IPA website (https://qiagen.my.salesforce-sites.com/KnowledgeBase/articles/Knowledge/Legend)

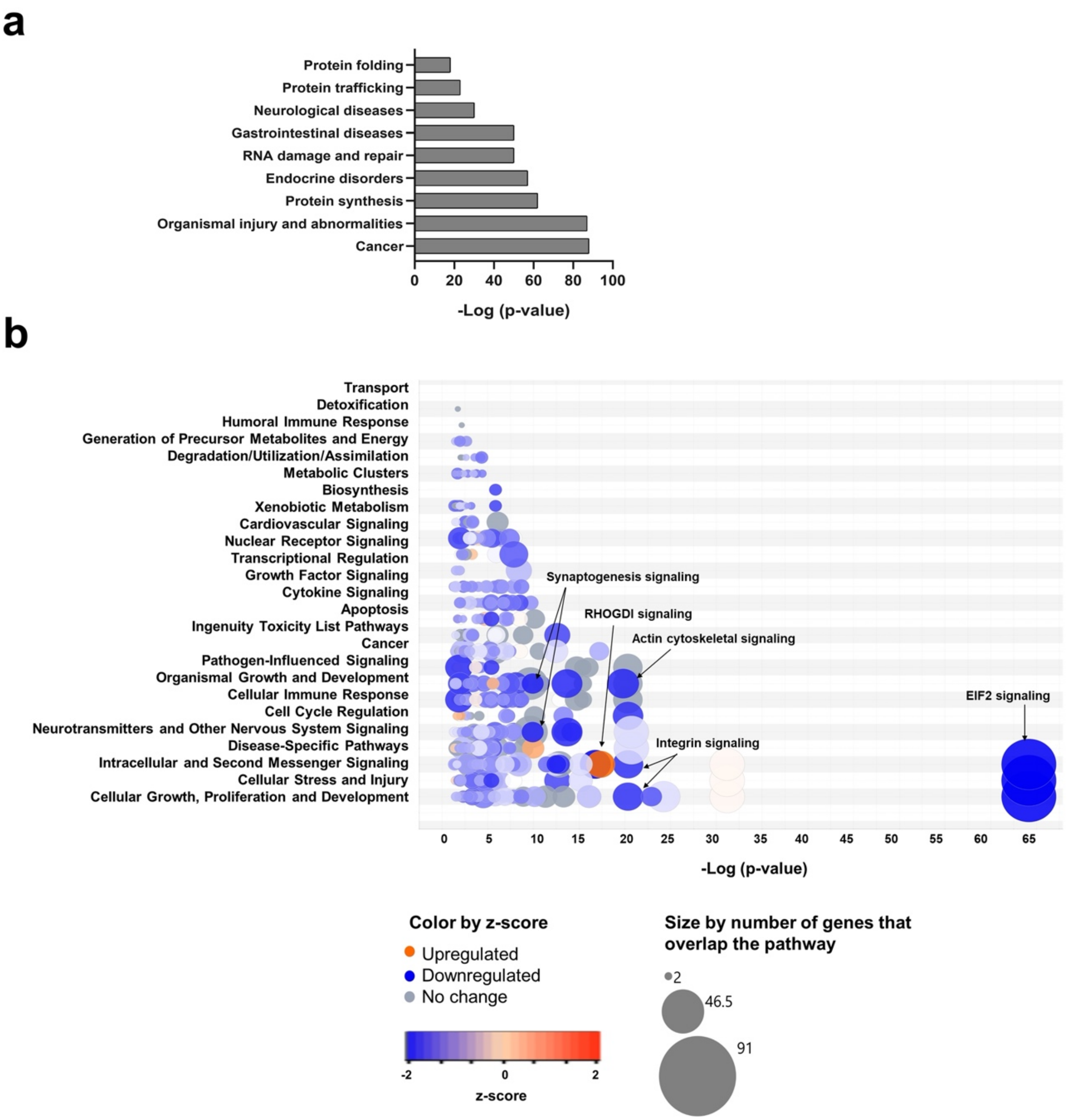

Fig. S8 Qiagen IPA disease and function analysis of proteomics results (a) The bar chart depicts relevant biological processes and diseases predicted to have significant alterations in MDS according to ingenuity ontology. All p-values calculated using Fischer's exact test p-value cut-off of 0.05 and range of $1.4 < \log_2$ fold change > -1.4. **(b)** Bubble chart depicting multiple pathway categories associated with top canonical pathways (see **Fig. 2f**) having significant -log (p-value) (EIF2 signaling, RHOGDI signaling, synaptogenesis signaling etc.). The size of the bubble corresponds to the number of genes involved in the overlapping pathways. Activated nodes with a positive z-score ≥ 2 are orange, inhibited nodes with a negative z-score ≤ -2 are blue and pathways lacking a significant activity pattern are gray

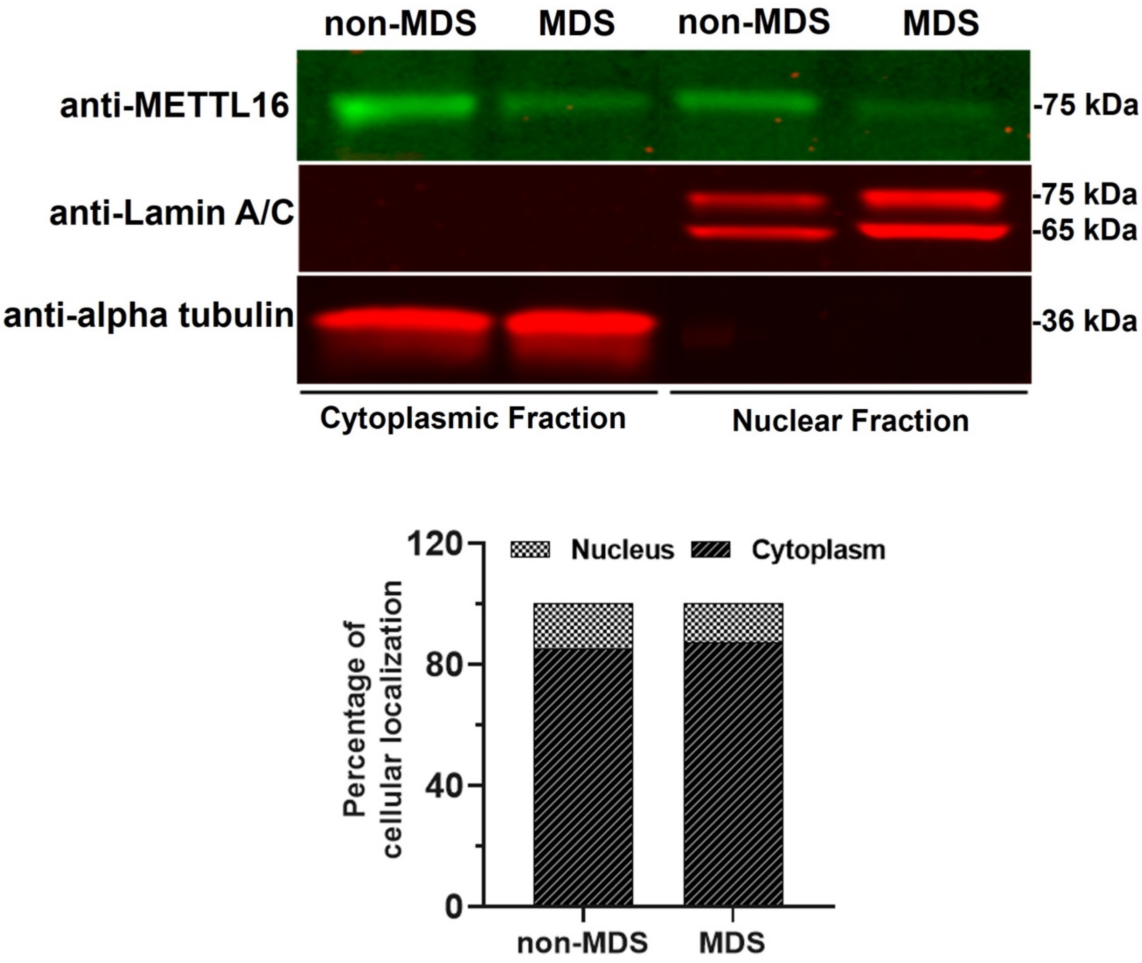

Fig. S9 Subcellular localization of human METTL16. Lamin A/C and α-tubulin are controls for the nuclear and cytoplasmic fractions, respectively. Cytoplasmic and nuclear METTL16 are shown as a percentage of total METTL16 in a stacked bar plot for non-MDS and MDS cells. Cytoplasmic fractions were normalized to α-tubulin, and the nuclear fractions were normalized to Lamin A/C

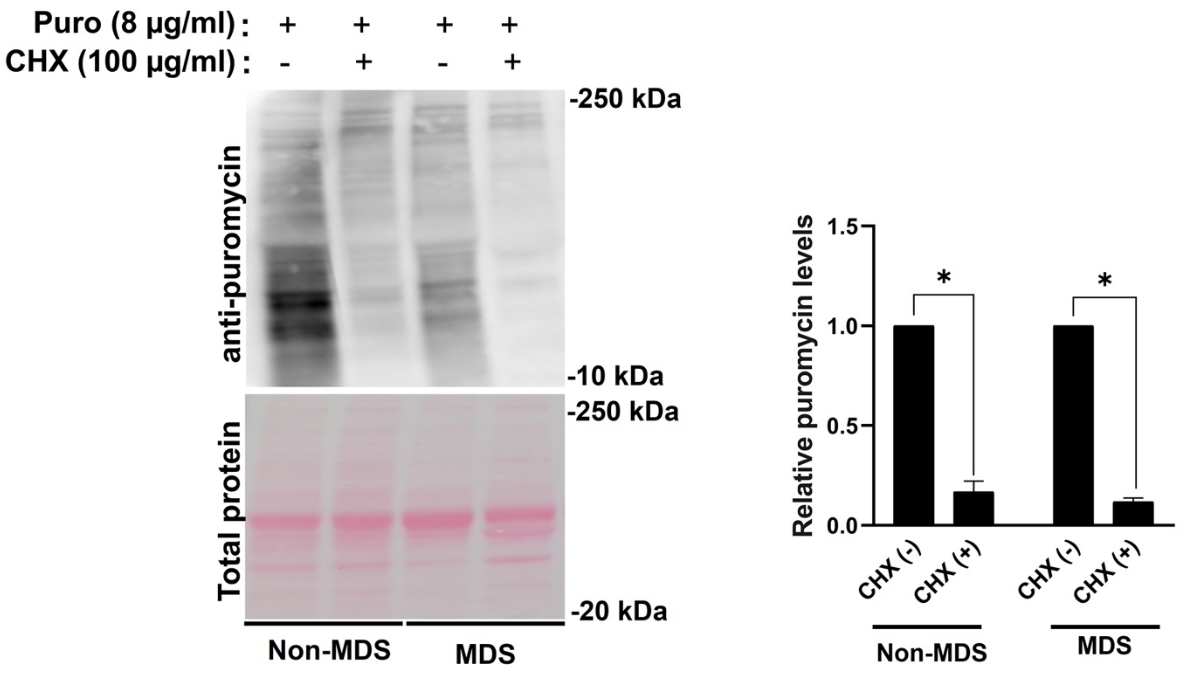

Fig. S10 Global levels of translation using SUnSET assay. Incorporation of puromycin was visualized via western blot for cells treated with or without 100 µg/ml cycloheximide (CHX). Total protein levels were visualized via Ponceau S staining. Quantification of puromycin is shown in the bar graph and values represent averages ± standard deviation of three biological replicates (n=3). *p-value is < 0.02 using a two-tailed unpaired student *t*-test

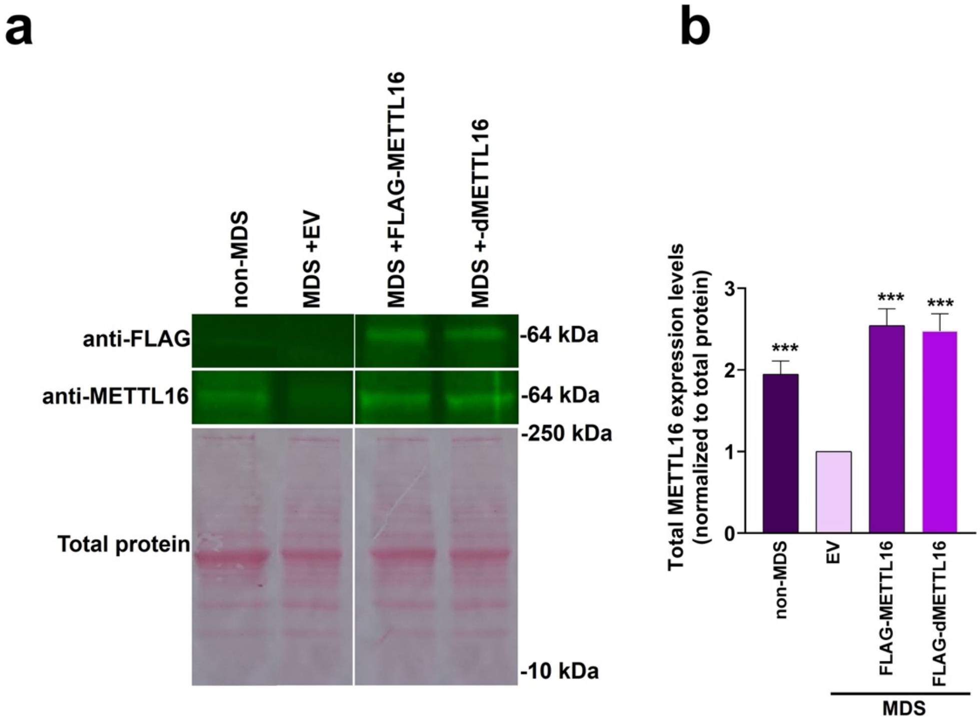

Fig. S11 Expression levels of METTL16 in MDS cells and MDS cells overexpressing FLAG-METTL16. (a) Western blot probed using anti-FLAG and anti-METTL16 antibodies in non-MDS cells, MDS cells transfected with empty vector or MDS cells transfected with WT FLAG-METTL16 and FLAG-dMETTL16. Ponceau S staining of total protein is shown as a loading control. **(b)** Quantification of total METTL16 levels after normalization to the total protein levels. MDS+EV was set an arbitrary value of 1.

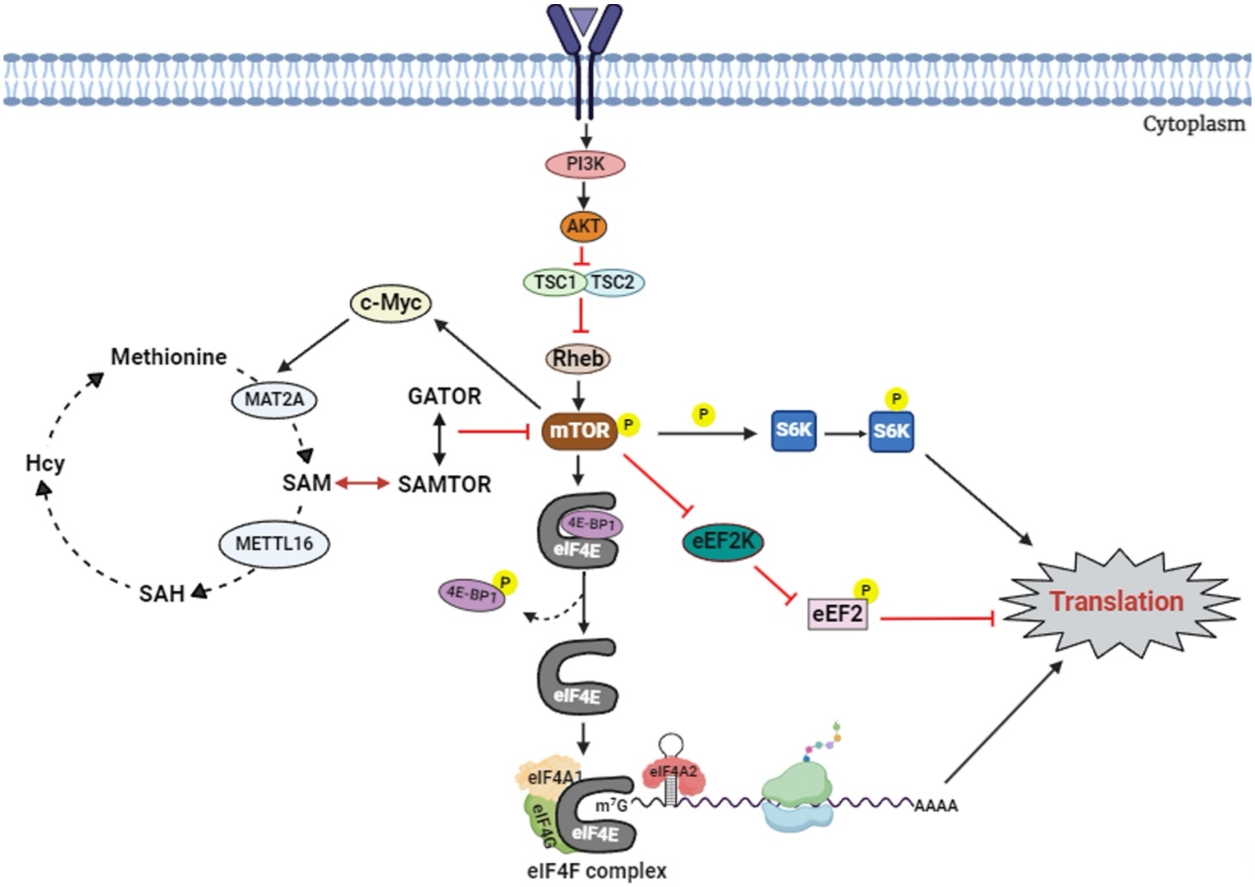

Fig. S12 mTOR signaling modulates SAM homeostasis. Schematic showing the methionine cycle and mTOR pathway, highlighting the critical factors. mTOR signaling stimulates SAM synthesis via the regulation of MAT2A levels in the methionine cycle. SAMTOR, which acts as a SAM sensor, hinders the mTOR signaling by engaging with GATOR. Direct interaction of SAM with SAMTOR disrupts the SAMTOR-GATOR complex. Under low cellular levels of SAM/SAH, the interaction between SAMTOR and GATOR inhibits the mTOR signaling in a SAMTOR-dependent manner. However, in the presence of SAM/SAH, the SAM-SAMTOR interaction disrupts the SAMTOR-GATOR dimer, activating mTOR signaling and hence protein translation. Thus, SAMTOR connects SAM homeostasis to mTOR signaling in protein translation. This schematic was created using BioRender [1].

Table S1: Primer sequences (5' to 3') used in the quantitative real-time PCR assay			
**Gene name**	**Gene description**	**Forward primer**	**Reverse primer**
ADD2	Adducin 2	TGAAGGCTGACGAGGTGGAG	GCTCTTCTCGGCAATGACGC
APOE	Apolipoprotein E	GCGTTGCTGGTCACATTCCT	AAAAGCGACCCAGTGCCAGT
BEX1	Brain Expressed X-Linked 1	TCGAGAATCGGGAGGAGGAGAC	GCCAAGGGCTCCCCTTTATTAG
CAMK2B	Calcium/calmodulin-Dependent Protein Kinase II Beta	CTGTGGACATCTGGGCATGT	GGATGGCTCCCTTGAGCTTT
CACNG4	Calcium Voltage-Gated Channel Auxiliary Subunit Gamma 4	ACTACGACCACGACAGCTCG	TGTAGATCCTGCCAGCACCG
CDH2	Cadherin-2	CCTACTGGACGGTTCGCCAT	TCTGCAGCGTTCCTGTTCCA
CRK	CRK Proto-Oncogene	ATTGGCAATTGGAGGCGGTA	TGGAACGGAACAGTCTGTCG
DLG1	Discs Large MAGUK Scaffold Protein 1	CCTTCTTGGGCCAGACACCA	GAAACCAAGGCCCGTTGAGC
GABBR2	Gamma-Aminobutyric Acid Type B Receptor Subunit 2	AGCAGATCCGCAACGAGTCA	GGAGCTCAGGGGCTCGAAAT
GJA5	Gap Junction Protein Alpha 5	ACAACACAAGGCAGCAAGCA	CTTCTCCTGCATGCGCACAG
GRIA1	Glutamate Ionotropic Receptor AMPA Type Subunit 1	GGACGCTGGAAGCAGGATCT	TGGACTCCAGGAGGTAGGCA
HAND2	Heart and Neural Crest Derivatives Expressed 2	AGCCCCCTCTGCGGC	GGTGGGGAAAACCACCTACC
HIC1	HIC ZBTB Transcriptional Repressor 1	TCGGTGACAACCTGTACGTG	ACTTCTTCCCGCAGATGGTG
INPP5K	Inositol Polyphosphate-5-Phosphatase K	ATTGGTTTGCAGGAATTGAACT	GGAAACTGCTCCACGAGTCA
KCNN2	Potassium Calcium-Activated Channel Subfamily N Member 2	GCAGCTGCCAATGTACTCAGG	CTTATGAGCCCAGGGAGGGC
MAT2A (spliced)	Methionine Adenosyltransferase II, Alpha	GCTCCTTCGTAAGGCCACTT	AAAGGAATGTGCCCTCCTCG
MAT2A (IR isoform)	Methionine Adenosyltransferase II, Alpha	CCACCCAGATAAGATTTGTGACC	GATGTAATTTCCCCAGCAAGAAG
METTL16	Methyltransferase 16, RNA N^6-Adenosine	GAACCTCGGCCTCAGAGATG	GCAAAGTCAGGAGGTTTGTCC
MNT	MAX Network Transcriptional Repressor	TGCAAAGCCCTTTCTTGTCG	AGCGTCTCTATGCTCATCGC
MYO1C	Myosin IC	CAATAGGAAAGCCAGGGCCA	GCTGGGCTGGATGATCTCTC
NRXN3	Neurexin 3	GTACGCTTCACCAGGAACGG	CTGGTGGCATGGAGGTCGTC
OVCA2	Ovarian Tumor Suppressor Candidate 2	GCTTCCGTGAGAAGACCGGG	CAATGCGGAGAAAACGTCGG
PAFAH1B1	Platelet Activating Factor Acetylhydrolase 1b Regulatory Subunit 1	GATGGGAGTGAAGGACGGAA	GTGCCATCTTGATTTGGCCG
PITPNA	Phosphatidylinositol Transfer Protein Alpha	TGGTGCTGCTCAAGGAGTAT	AATGGCCTGTCACTTGGGAG

PRPF8	Pre-MRNA Processing Factor 8	GAAAGCGGCCTCTTGTGTGA	CCGACATGTAGTCCGGTAGC
RILP	Rab Interacting Lysosomal Protein	CCTGCTCAAGGAGGAACTGG	CTCCCTTGTCATCGGAGAGC
RTN4RL1	Reticulon 4 Receptor-Like 1	CCCCAGACTACCAGCACAAG	TCCAGACCTCTCGGAACGAT
SEMA4D	Semaphorin 4D	GCATTTGCACCCATACCCCG	AAAGGGAAGTGGCGCTGAGT
SLC43A2	Solute Carrier Family 43 Member 2	CTACACCTCCATCTTCGGCG	TGGTTGGCGTCTTTCTCCTC
SERPINF1	Serpin Family F Member 1	AGCTGCGCATAAAATCCAGC	GGCGAGCTTCCCTTTCATCT
SMG6	SMG6 Nonsense Mediated mRNA Decay Factor	CTGCTCCCTCTCTCCTCACT	TCCACGCCTGGAAGTTTCTC
SMYD4	SET and MYND Domain Containing 4	TCTGCCTGTGGATGAATGGA	TCCCTCAAGGTCTCTGCTGT
SNCA	Synuclein Alpha	AACGTGTCACGCTGCAGAAC	CTTGGTCGGTCAGCCTTCCT
ST8SIA2	ST8 Alpha-N-Acetyl-Neuraminide Alpha-2,8-Sialyltransferase 2	GATTGACGCCCACAGCTTCG	AGGATGCTGCCATTGAGGCT
STX1A	Syntaxin 1A	TGACACCAAGAAGGCCGTCAA	AACAGTGGAGGCGATGACGA
SYT7	Synaptotagmin 7	CCCATTGGGGAGGTGTCCAT	TCTTCTCCACCCGCTTGTCC
THBS2	Thrombospondin 2	GGATGTACGTGGCCAAAGGC	TACTCGGTGGTGACATGCGG
WDR81	WD Repeat Domain 81	CGGCTTCTCCTCAGGCTTC	GGACTCCCCCACTCCATTTG
WNT16	Wnt Family Member 16	TCCCCATCGGAAACACCACG	CAGCACAGGAGCCGGAAACT
YWHAE	Tyrosine 3-Monooxygenase/Tryptophan 5-Monooxygenase Activation Protein Epsilon	TTACTGTGTCGTCTCCGTGC	AACGAAGTCCCTCCCCAAAC

Table S2: Primary antibodies used in western blots				
**Primary antibody (RRID)**	**Gene description**	**Species and clonality**	**Manufacturer**	**Dilutions**
4e-BP1 (AB_331692)	4e-Binding Protein	Rabbit Polyclonal	Cell Signaling Technology (CST)	1: 1000
phos-4e-BP1 (AB_560835)	Phosphor 4e-Binding Protein	Rabbit Monoclonal	Cell Signaling Technology (CST)	1: 1000
AHNAK (AB_1839460)	Neuroblast Differentiation-Associated protein	Mouse Monoclonal	Sigma-Aldrich	1-5 µg/mL
ARSA/ASA (AB_2060420)	Arylsulfatase A	Mouse Polyclonal	abcam	1:500
CNN1/ Calponin I (AB_2291941)	Calponin 1	Rabbit Monoclonal	abcam	1:5000
CAMK2B (AB_1845941)	Calcium/Calmodulin Dependent Protein Kinase II Beta	Rabbit Polyclonal	Thermo Fisher Scientific	0.4 µg/mL
CBS (AB_11155108)	Cystathionine Beta-Synthase	Rabbit Monoclonal	abcam	1:1000
CRK (AB_10738692)	CRK Proto-Oncogene, Adaptor Protein	Mouse Polyclonal	Sigma-Aldrich	1 µg/mL
FBLN1/ Fibulin (AB_2925194)	Fibulin 1	Rabbit Monoclonal	abcam	1:1000
FLAG (AB_1957945)	N/A	Mouse Monoclonal	Thermo Fisher Scientific	1:1000
FXR1 (AB_11154960)	FMR1 Autosomal Homolog 1	Rabbit Monoclonal	abcam	1:1000
GOPC (AB_10860292)	Golgi Associated PDZ and Coiled-Coil Motif Containing	Rabbit Monoclonal	abcam	1:1000
MAT2A (AB_2608990)	Methionine Adenosyltransferase II, Alpha	Rabbit Polyclonal	Thermo Fisher Scientific	1:1000
METTL16 (AB_1853828)	Methyltransferase 16, RNA N^6-Adenosine	Rabbit Polyclonal	Sigma-Aldrich	1 µg/mL
mTOR (AB_330978)	Mammalian Target of Rapamycin	Rabbit Polyclonal	Cell Signaling Technology (CST)	1: 1000
phos-mTOR (AB_330970)	Phosphor-Mammalian Target of Rapamycin	Rabbit Polyclonal	Cell Signaling Technology (CST)	1: 1000
MYO1C (AB_10668604)	Myosin IC	Rabbit Polyclonal	Sigma-Aldrich	1 µg/mL
PAFAH1B1 (AB_1842816)	Platelet Activating Factor Acetylhydrolase 1b Regulatory Subunit 1	Mouse Monoclonal	Sigma-Aldrich	1 µg/mL
Puromycin (AB_2566826)	N/A	Mouse Monoclonal	EMD Millipore	1:12500
P70 S6K (AB_331676)	P70 S6 Kinase	Rabbit Polyclonal	Cell Signaling Technology (CST)	1: 1000
phos-P70 S6K (AB_330944)	Phosphor-P70 S6K	Rabbit Polyclonal	Cell Signaling Technology (CST)	1: 1000
SERPINF1 (AB_1856733)	Serpin Family F Member 1	Rabbit Polyclonal	Sigma-Aldrich	1 µg/mL

SHANK2 (AB_2925199)	SH3 and Multiple Ankyrin Repeat Domains 2	Mouse Monoclonal	abcam	1:1000
SPTAN1/ NEAS (AB_298540)	Spectrin Alpha, Non-Erythrocytic 1	Mouse Monoclonal	abcam	1:1000
TPM4 (AB_971894)	Tropomyosin 4	Rabbit Polyclonal	abcam	1:1000
TRAM-1 (AB_1930654)	Translocation-Associated Membrane Protein 1	Rabbit Polyclonal	abcam	1:500
YWHAE (AB_10667572)	Tyrosine 3-Monooxygenase/Tryptophan 5-Monooxygenase Activation Protein Epsilon	Rabbit Polyclonal	Sigma-Aldrich	1 µg/mL

Table S3: Secondary antibodies used in western blots			
**Secondary antibody (RRID)**	**Species and clonality**	**Manufacturer**	**Dilutions**
Goat anti-Rabbit IgG (H+L) Highly Cross-Adsorbed Secondary Antibody, Alexa Fluor™ Plus 555 (AB_2633281)	Rabit Polyclonal	Thermo Fisher Scientific	0.4 µg/mL (1:5000)
Goat anti-Mouse IgG (H+L) Highly Cross-Adsorbed Secondary Antibody, Alexa Fluor™ Plus 647 (AB_2633277)	Mouse Polyclonal	Thermo Fisher Scientific	0.2 µg/mL (1:10000)
Rabbit anti-Mouse IgG, HRP conjugate (AB_92531)	Rabbit Polyclonal	Sigma-Aldrich	1:2000

Table S4: Primer sequences (5' to 3') for cloning and site-directed mutagenesis

Wild-type FLAG-METTL16 (1-562)	Forward<br>HindIII site + FLAG tag	GTTTAAACTTAAGCTTATGGACTACAAAGACGATGACGACAAG<br>GCTCTGAGTAAATCAATGCATG
	Reverse<br>NotI site	TAGACTCGAGCGGCCGCGTTAACTGCAACAAGCCTG
FLAG-dMETTL16 (1-562)	R82A forward	CCCACAGTTCCCTTGGCACTCAACTATATTCACTGG
	R82A reverse	CCAGTGAATATAGTTGAGTGCCAAGGGAACTGTGGG
	N184A forward	GACTTTTGCATGTGCGCCCCTCCCTTTTTTGCCAATC
	N184A reverse	GATTGGCAAAAAAGGGAGGGGCGCACATGCAAAAGTC

Table S5: RNA nucleoside modifications examined from non-MDS and MDS cells	
**Nucleoside**	**Nucleoside symbol**
Adenosine	A
Guanosine	G
Cytidine	C
Uridine	U
2'-*O*-methyladenosine	Am
N^1-methyladenosine	m^1A
2,8-dimethyladenosine	$m^{2,8}A$
N^6-isopentenyladenosine	i^6A
N^6-methyladenosine	m^6A
N^6,2′-*O*-dimethyladenosine	m^6Am
N^6,N^6-dimethyladenosine	$m^{6,6}A$
N^6-threonylcarbamoyladenosine	t^6A
2-methylthio-N^6-threonylcarbamoyladenosine	ms^2t^6A
N^6-methyl-N^6-threonylcarbamoyladenosine	m^6t^6A
2-methylthio-N^6-isopentenyladenosine	ms^2i^6A
Inosine	I
1-methylinosine	m^1I
2′-*O*-methylguanosine	Gm
1-methylguanosine	m^1G
2-methylguanosine	m^2G
N^2,N^2-dimethylguanosine	$m^{2,2}G$
N^2,$N^{2,7}$-trimethylguanosine	$m^{2,2,7}G$
7-methylguanosine	m^7G
Queuosine	Q
8-oxoguanine	8-oxoG
Hydroxywybutosine	OHyW
2'-*O*-methylcytidine	Cm
3-methylcytidine	m^3C
N^4-acetylcytidine	ac^4C
5-methylcytidine	m^5C
2'-*O*-methyluridine	Um
Pseudouridine	ψ
Dihydrouridine	D
3-methyluridine	m^3U
5-methyluridine	m^5U
5-aminomethyluridine	nm^5U
5-carbamoylmethyl-2'-*O*-methyluridine	ncm^5Um

Reference

1. BioRender (2023) Accessed: May 7, 2023. https://www.biorender.com/.
